# Upregulated miRNAs on the *TP53* and *RB1* Binding Seedless Regions in High-Risk HPV-Associated Penile Cancer

**DOI:** 10.3389/fgene.2022.875939

**Published:** 2022-06-24

**Authors:** Jenilson da Silva, Carla Cutrim da Costa, Ingryd de Farias Ramos, Ana Carolina Laus, Luciane Sussuchi, Rui Manuel Reis, André Salim Khayat, Luciane Regina Cavalli, Silma Regina Pereira

**Affiliations:** ^1^ Postgraduate Program in Health Science, Federal University of Maranhão, São Luís, Brazil; ^2^ Degree in Biological Sciences, Department of Biology, Federal University of Maranhão, São Luís, Brazil; ^3^ Postgraduate Program in Oncology and Medical Sciences, Federal University of Pará, Belém, Brazil; ^4^ Molecular Oncology Research Center, Barretos Cancer Hospital, Barretos, Brazil; ^5^ Oncology Research Center, Federal University of Pará, Belém, Brazil; ^6^ Institute of Biological Sciences, Federal University of Pará, Belém, Brazil; ^7^ Research Institute Pelé Pequeno Príncipe, Faculdades Pequeno Príncipe, Curitiba, Brazil; ^8^ Laboratory of Genetics and Molecular Biology, Department of Biology, Federal University of Maranhão, São Luís, Brazil

**Keywords:** penile cancer, tumor suppressor repression, miRNA, HPV, *TP53*, *Rb1*

## Abstract

Cancer development by the human papillomavirus (HPV) infection can occur through the canonical HPV/p53/RB1 pathway mediated by the E2/E6/E7 viral oncoproteins. During the transformation process, HPV inserts its genetic material into host Integration Sites (IS), affecting coding genes and miRNAs. In penile cancer (PeCa) there is limited data on the miRNAs that regulate mRNA targets associated with HPV, such as the *TP53* and *RB1* genes. Considering the high frequency of HPV infection in PeCa patients in Northeast Brazil, global miRNA expression profiling was performed in high-risk HPV-associated PeCa that presented with *TP53* and *RB1* mRNA downregulated expression. The miRNA expression profile of 22 PeCa tissue samples and five non-tumor penile tissues showed 507 differentially expressed miRNAs: 494 downregulated and 13 upregulated (let-7a-5p, miR-130a-3p, miR-142-3p, miR-15b-5p miR-16-5p, miR-200c-3p, miR-205-5p, miR-21-5p, miR-223-3p, miR-22-3p, miR-25-3p, miR-31-5p and miR-93-5p), of which 11 were identified to be in HPV16-IS and targeting *TP53* and *RB1* genes. One hundred and thirty-one and 490 miRNA binding sites were observed for *TP53* and *RB1*, respectively, most of which were in seedless regions. These findings suggest that up-regulation of miRNA expression can directly repress *TP53* and *RB1* expression by their binding sites in the non-canonical seedless regions.

## Introduction

The *TP53* gene is known as the “sentinel gene” due to its ability to identify cell damage and to coordinate complex mechanisms to mediate cell repair, protecting genome stability and, consequently, cell homeostasis. Therefore, it is not surprising that *TP53* tumor suppressor is the most frequently mutated gene in human tumors. Most of the alterations described are missense mutations, whereby the protein loses its primary function, or it acquires oncogenetic functions (Datta et al., 2017; Wang and Sun, 2017; Sammons et al., 2020). In addition, the p53 mutant protein may also facilitate the adaptation of tumor cells to the disadvantageous environment that arises as the tumor grows (Mantovani et al., 2019).

The association between human papillomavirus (HPV) and some cancers, including cervical, head and neck, vulvar, anorectal, and penile squamous cell carcinomas (SCC), is well characterized by the canonical mechanism involving the HPV oncogenes E6 and E7 and p53 and RB1 proteins (de Martel et al., 2017). During the transformation process, HPV inserts its genetic material into host human integration sites (IS), which have been identified in regions harboring cancer-related genes, as well as in regions presenting copy number alterations (CNAs) (Busso-Lopes et al., 2015; Macedo et al., 2020; Pinatti et al., 2021). Indeed, integration of DNA-copy number alterations and other omics data have shown that DNA methylation, mRNA, and miRNA expressions alterations affect coding-genes and miRNAs located within or near the HPV common integration sites (Barzon et al., 2014; Groves and Coleman, 2018; Rosa et al., 2019; Pinatti et al., 2021).

In the last decade, it has been also demonstrated that the wild-type p53 protein plays primarily its role as a transcription factor by regulating a large network of protein-coding genes and non-coding RNAs, including miRNAs, both inducing or repressing their targets (Hermeking, 2012; Fischer, 2017). In addition, the p53 protein regulates miRNA processing, primarily through its central DNA-binding domain, a target site of most cancer-specific mutations. Interestingly, miRNAs can also regulate p53 expression by matching in the seed region into the 3′UTR of *TP53* mRNA, directly inducing the repression of *TP53* or its regulators (Hermeking, 2012; Hermeking et al., 2014). Although several tumor-specific alterations in the p53-miRNA network have been described in different cancers (Hermeking, 2012; Datta et al., 2019), there is no data on miRNAs targeting of *TP53* gene in HPV-associated penile cancer.

PeCa is a rare carcinoma in developed countries, but it presents higher incidence rates in South America, Asia, and Africa, where limited economic and social conditions play a large impact leading to delay in diagnosis, and treatment initiation. In Brazil, specifically in the Northeast region that is particularly affected by low socio-economic conditions and educational levels and high frequency of HPV infection, presents a high incidence of PeCa, with patients presenting additional comorbidities, which contributes to a high incidence of mortality rates (Macedo et al., 2020; Silva et al., 2021) However, even in countries that are not impacted by major economic limitations, the incidence and mortality rates of PeCa has increased, mainly among younger patients (Hansen et al., 2018). Hence, the increased occurrence of PeCa, irrespectively of the countries’ socioeconomic conditions, has suggested that HPV infection is possibly the main triggering mechanism for tumor development, in addition to poor hygiene of the genital region, phimosis, uncircumcision, and chronic inflammation (Christodoulidou et al., 2015; Kidd et al., 2017; Adashek et al., 2019).

PeCa treatment options are limited. No effective target therapy is available, mainly due to the scarcity of knowledge on the molecular pathways involved in the development and progression of these tumors. Limited data is available on the role and mechanisms of miRNA deregulation in PeCa, including those that disrupt miRNAs targets that regulate critical genes associated with the action of HPV, such as the *TP53* and *RB1* genes. Considering the high frequency of HPV infection in patients with advanced PeCa in Maranhão State, in Northeast of Brazil, in the present study miRNAs expression analysis was performed in high-risk HPV-associated PeCa with *TP53* and *RB1* mRNA downregulated expression, as previously reported by our group in >80 and 60% of the patients, respectively (Macedo et al., 2020).

This study opens the opportunity to better understand the role of *TP53* and *RB1* transcriptional regulators in HPV-associated penile carcinomas and brings much needed knowledge on the molecular tumorigenesis of this still-neglected tumor.

## Materials and Methods

### Sample Cohort

Fresh PeCa chemotherapy-naive surgical resection tissue specimens were obtained from 22 patients from the Aldenora Hospital, São Luís, Maranhão, Brazil. These patients are a subset of a larger cohort of 37 patients previously investigated for HPV *status*, gene, and protein expression for *TP53* and *RB1* (Macedo et al., 2020). All the samples were collected under patients’ written informed consent, approved by the Research Ethics Committee on Humans from the Federal University of Maranhão and by the National Research Ethics Commission (CONEP-Brazil, CAAE: 46371515.5.0000.5087). Tumor and adjacent non-tumor tissues, sampled from 2 cm distant from the tumor site after histopathological assessment, were obtained before any cancer treatment. At the time of the sample collection, the patients had no history of other cancers or sexually transmitted diseases.

The clinical and histopathological variables were obtained from patients’ medical records. The mean age of the patients at diagnosis was 64.22 ± 15.63 years, ranging from 32 to 85 years old. The patients declared themselves smokers (41%) and alcoholics (45.5%). All tumors were classified as squamous cell carcinoma (SCC), and the condylomatous and keratinized histological subtypes localized mostly in the glans, corpus cavernosum, and corpus spongiosum were the most frequent, 45.4 and 36.4%, respectively. Tumor grades II and III were the most frequent, present in 54.5 and 27.3% of the patients, respectively. Lymphatic and perineural invasion were positive in 18.2 and 22.8% of the patients, who presented mostly ulcerated lesions (68.2% of the cases), followed by vegetative (18.2%) and verrucous (13.6%). Penectomy (partial and total) was performed in 95.4% of the patients. The primary tumor of each patient was positive for HPV by Nested-PCR and DNA sequencing, as described in Macedo *et al.* In this subset, the multiple infections were detected in 50.0% of the cases. The HPV16 genotype was the most frequent (72.2%), followed by the 74 (16.6%), 30, 59 and 66 (11%, each) genotypes. Genotypes found in lower frequencies were 6, 18, 30, 35, 44, 53, 58, and 73 (Supplementary Table S1). Four cases were positive for HPV, but not genotyped since the samples did not have sufficient DNA for Nested-PCR and/or DNA sequencing analysis. Table 1 and Supplementary Table S1 present the detailed patients’ clinical-histopathological information.

**TABLE 1:** Clinical-histopathological profile of patients diagnosed with HPV positive penile carcinoma (*n* = 22).

Variable	Number (%)	Variable	Number (%)
1. Histological subtype		5. Lesion	
Condylomatous	10 (45.4%)	Ulcerated	15 (68.2%)
Keratinized PeCa	8 (36.4%)	Vegetative	4 (18.2%)
Mixed	4 (18.2%)	Verrucous	3 (13.6%)
2. Tumor size		6. Tumor site	
0.6–2.0	4 (18.2%)	Glans	9 (40.9%)
2.1–5.0	15 (68.2%)	Glans and foreskin	7 (31.8%)
5.1—10.0	3 (13.6%)	Foreskin	2 (9.1%)
3. Tumor stage		Glans, foreskin and other areas	4 (18.2%)
pT1	6 (27.3%)	7. Surgery type	
pT2	9 (40.9%)	Preserved penis	1 (4.5)
pT3	7 (31.8%)	Partial penectomy	16 (72.7%)
4. Tumor grade		Radical penectomy	5 (22.7%)
I	4 (18.2%)	8. Phimosis occurrence	
II	12 (54.5%)	Yes	8 (36.3%)
III	6 (27.3%)	No	9 (41.0%)
—	—	No information	5 (22.7%)

Considering our previous study (Macedo et al., 2020) in which we have demonstrated *TP53* and *RB1* down-regulated expression at both mRNA (by real-time PCR) and protein (by immunohistochemistry) levels (86 and 65% of the cases, respectively), in this study, we investigated the possible mechanisms by which these genes might be repressed in HPV-associated PeCa. For that, a subset of 22 tumors was evaluated for differential miRNA expression in relation to adjacent non-tumor tissues (*n* = 5). Fifteen of the 22 tumors have data on the expression of *TP53* and *RB1* (73 and 69% of the tumors are underexpressed, respectively) (Supplementary Table S1). Subsequently, prediction miRNAs binding sites analysis were performed in the *TP53* and *RB1* gene, followed by a search for molecular pathways potentially involved in penile carcinogenesis in HPV-positive patients.

### Global miRNA Expression Analysis

Total RNA from 22 PeCa tumors and five adjacent non-tumor tissues was isolated using the TRIzol protocol (Invitrogen Carlsbad, CA, United States). RNA concentration and quality were tested by measuring the 260/280 and 260/230 ratios using the Nanodrop 2001 spectrophotometer (Willington, DE, United States). Expression of miRNAs was determined using the *nCounter® Human v.3 miRNA expression* platform (*Nanostring Technologies™*, Seattle, Wa, United States), which contains human probes from miRBase v.22 (http://www.mirbase.org) targeting 827 human miRNAs, six positive controls, eight negative controls, three positive binding controls, three negative binding controls, five internal reference genes (*ACTB*, *B2M*, *GAPDH*, *RPL19,* and *RPL0*) and five miRNA controls (ath-miR- 159a, cel-miR-248, cel-miR-254, osa-miR-414, and osa-miR-442) as previously reported at the Molecular Oncology Research Center (Pessôa-Pereira et al., 2020; Causin et al., 2021). The raw data were pre-processed and exported as RCC files. The raw data of the study, as well as the clinical information of the patients are available for access from the *Gene Expression Omnibus* (GEO), under registration GSE197121.

### Differential miRNA Expression Analysis

The raw data were normalized and analyzed using the ROSALIND^®^ Nanostring platform (https://rosalind.onramp.bio/). Adjacent non-tumor tissues distant 2 cm for the primary tumor were used as control. Read distribution percentages, identity heatmaps, and sample MDS plots were generated as part of the QC step. The normalization was conducted following the background subtraction based on POS_A probes correction factors (positive control normalization and codeset normalization). For both steps, the geometric mean of each probeset was used to create a normalization factor. The fold changes, *p*-values for comparisons were calculated using the *t*-test method (*p* ≤ 0.05). *p*-value adjustment was performed using the Benjamini–Hochberg (*p* ≤ 0.01) method to estimate false discovery rates (FDR). The clustering of miRNAs for the final heatmap was constructed using the PAM (Partitioning Around Medoids) through a method using the FPC R library (Hennig, 2020) that takes into account the direction and type of all signals in a pathway, the position, function, and type of each miRNA identified. Fold change (≥2 for miRNAs upregulated and ≤-2 for miRNAs downregulated), *p*-value and adjusted *p*-value were used as selection criteria for miRDE.

### Prediction of miRNA Binding Sites in the *TP53* and *RB1* Gene Sequences

The *STarMir* software (Kanoria et al., 2016) was used to identify the miRNAs binding regions in *TP53* and *RB1* genes (CLIP-data). The construction design and nucleic acid fold of *STarMir* are obtained from the *Mfold* package (Zuker, 2003) and *Sfold* which contains the *Srna* module (Ding et al., 2004). *Sfold* applies a two-step model for hybridization between mRNA and miRNA. In this model, hybridization of the miRNA-target occurs at an accessible target site and then the hybrid elongates to form the complete miRNA-target duplex. The minimum free energy of hybridization was obtained from the RNA*hybrid* tool (Rehmsmeier et al., 2004; Long et al., 2007). Only interactions in “seed” and “seedless” regions with LogitProb values ≥0.5; ΔG_hybrid_ ≤ -10.00 and site-access ≥ 0.4 were considered.

### Pathway’s Enrichment Analysis

Pathway’s enrichment analysis was performed by miRPath v.3 - DIANA TOOLS software (Vlachos et al., 2015) using the Tarbase prediction algorithm and considering the *p*-value threshold ≤0.05. The generated pathways are part of the *Kyoto Encyclopedia of Genes and Genomes* (KEGG). The *TP53* and *RB1* genes were used as filters to generate KEGG pathways. The *‘pathways union’* function was used to generate the related top pathways, considering the *p*-value threshold ≤0.05 and enrichment analysis method by Fisher’s Exact Test.

## Results

### Overexpressed miRNAs Targeting TP53 and RB1 in Penile Cancer Patients

**FIGURE 1:**
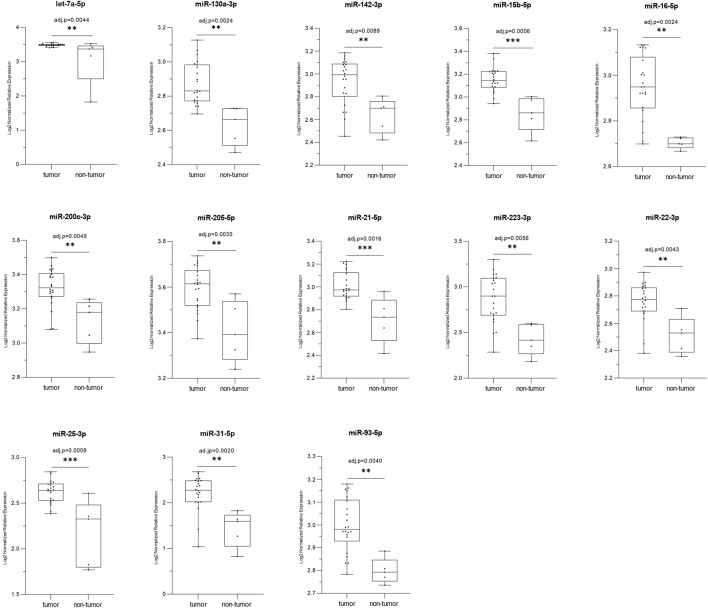
The box-plots represent a comparison of the relative expression levels of the 13 miRDE upregulated in tumors (*n* = 22) versus normal tissue (*n* = 5); *p* ≤ 0.05 by *t*-test and adj.-p ≤ 0.01 by Benjamini–Hochberg; *p ≤ 0.05, **p ≤ 0.01 and ***p ≤ 0.001.

Differential miRNA expression analysis was performed in the 22 PeCa tissues. The global miRNA expression profile of these tumors showed 507 differentially expressed miRNAs (miRDE) compared to a group of five adjacent non-tumor penile tissues. Among these miRDE, 494 (97.4%) miRNAs were downregulated and 13 (2.6%) upregulated (Figure 1; Table 2). Considering the previously detected lower *TP53* mRNA expression (85.7% (12/14) and lower protein expression in 87.5% (14/16) of these cases (Macedo et al., 2020), we further investigated the up-regulated miRNAs in the subset of 22 tumors, of which 73% were downregulated. The miRDEs let-7a-5p, miR-130a-3p, miR-15b-5p, miR-21-5p, and miR-25-3p were overexpressed in 100% of cases. Interestingly, 84.6% (*n* = 11) of miRDEs were found to be located at HPV integration sites. The HPV integration sites were identified as target regions of the oncogenic HPV16 genotype, the most frequently detected genotype in our study cohort (Supplementary Table S1).

**TABLE 2:** Thirteen differentially expressed miRNAs observed upregulated in the PeCa patients, and their respective chromosomal location and HPV integration sites (presented by miRNA number).

miRNAs	Cytoband	Start—Stop (bp)	Integration site HPV (genotype)^a^	miRNA expression	Frequency (%)	Log2FC	*p*-Value	Adj*.-p*
let-7a-5p	9q22.32	96,938,234–96,938,325	yes, (16.18)	upregulated	100.0	2.2266	0.0019	0.0044
miR-130a-3p	11q12.1	57,641,198–57,641,286	yes, (16)	upregulated	100.0	1.1603	0.0008	0.0024
miR-142-3p	17q22	58,331,222–58,331,327	yes, (16)	upregulated	86.4	1.4576	0.0054	0.0089
miR-15b-5p	3q25.33	160,404,588–160,404,685	yes, (16)	upregulated	100.0	1.6509	0.0000	0.0006
miR-16-5p	13q14.2	50,623,109–50,623,197	yes, (16)	upregulated	95.5	1.2512	0.0008	0.0024
miR-200c-3p	12p13.31	6,963,694–6,963,771	no	upregulated	95.5	1.2541	0.0023	0.0049
miR-205-5p	1q32.2	209,428,820–209,432,384	yes, (16.18)	upregulated	95.5	1.4610	0.0013	0.0035
miR-21-5p	17q23.1	59,841,262–59,841,342	yes, (16.18)	upregulated	100.0	1.4629	0.0004	0.0016
miR-223-3p	Xq12	66,018,870–66,018,979	no	upregulated	95.5	2.0222	0.0028	0.0056
miR-22-3p	17p13.3	1,617,197–1,617,281	yes, (16)	upregulated	91.0	1.0605	0.0018	0.0043
miR-25-3p	7q22.1	100,093,560–100,093,643	yes, (16)	upregulated	100.0	1.5448	0.0001	0.0008
miR-31-5p	9p21.3	21,512,114–21,512,184	yes, (16)	upregulated	91.0	2.0012	0.0006	0.0020
miR-93-5p	7q22.1	99,691,391–99,691,470	yes, (16)	upregulated	95.5	1.0525	0.0016	0.0040

a Data obtained from HPVBase (Kumar Gupta and Kumar, 2015) and VISDB (Tang et al., 2020).

Figure 1 Relative expression of thirteen miRNAs upregulated (tumor vs. non-tumor) in the PeCa studied.

**FIGURE 2:**
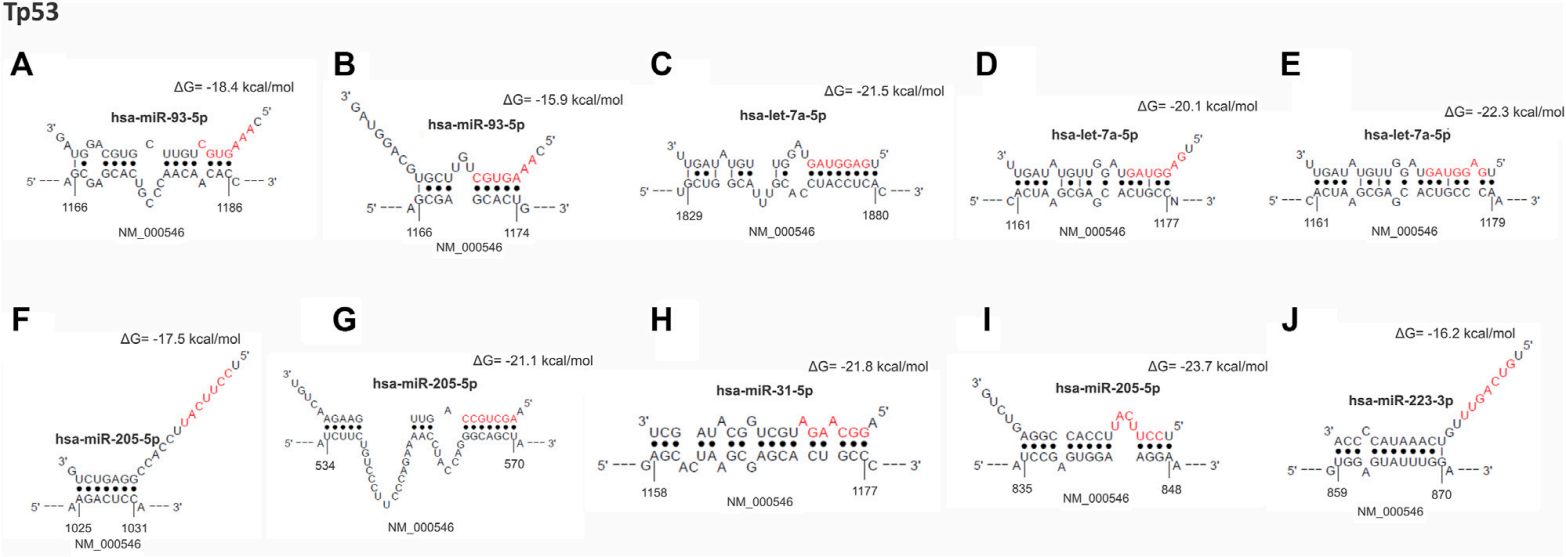
From **(A–J)**, hybrid mRNA (*TP53*)—miRNA upregulated. The upper and lower sequences represent the miRNA in the 3′-5′ sense and 5′-3′ sense hybridization sites in the mRNA, repectively. The numbers at the ends of the mRNA show the starting and ending position of the hybridization sites. Dots between the sequences indicate the paired nucleotides. Nucleotides in red mark the “seed” sequence in miRNA. The energy resulting from the hybridization was calculated by the RNA*hybrid* algorithm.

Prediction of miRNA binding sites revealed that all 13 up-regulated miRNAs targeted the *TP53* gene, acting as negative regulators of this tumor suppressor gene expression. We found 131 target sites for these miRNAs: 98.5% in the non-canonical seedless regions and two in the seed regions (Supplementary Table S2). Interestingly, *TP53* presents 129 seedless sites, in which all 13 differentially expressed miRNAs could bind. The coding region presented the highest number of target seedless regions with 81/129 sites (62.8%), followed by 3′UTR with 32/129 sites (24.8%) and 5′UTR with 16/129 sites (12.4%). Bindings in the gene seed regions were observed to occur with miR-22-3p and let-7a-5p and both interactions were of 8mer-type. The binding between let-7a-5p and *TP53* occurred in a canonical 3′UTR region, while the binding of miR-22-3p occurred in a non-canonical coding region (site position: 534–570 (bp)*;* seed position: 564–570 (bp). This region is highly conserved (site conservation = 0.963 and seed conservation = 0.933). Our analysis also revealed that miR-93-5p and let-7a-5p can bind to a higher number of seedless regions, 21 and 20 predicted binding sites, respectively, while miR-15b-5p, miR-16-5p, miR-223-5p, miR-22-5p, and miR-31-5p bind to a lower number of regions, i.e., five predicted sites for each. Considering the size of the *TP53* mRNA (2,591 bp; transcript variant 1, NCBI Reference Sequence: NM_000546.5) we observed that the 1,000–1073bp, 2,500–2580bp, and 835–899bp intervals are miRNA binding hotspots regions, harboring a total of 22, 16 and 14 sites, respectively. Figure 2; Table 3 show the ten main binding sites observed in the *TP53* gene.

**TABLE 3:** Top 10 miRNA binding regions identified in TP53 and RB1 genes.

Binding regions in TP53								
miRNA	Site position^a^	LogitProb^b^	Region	ΔG_hybrid_ ^c^	ΔG_total_ ^d^	Site access^e^	Site consv^f^	Site location^g^
miR-93-5p	1,166–1,174	0.864	CDS	−15.900	−15.316	0.714	0.959	0.820
miR-93-5p	1,166–1,186	0.848	CDS	−18.400	−18.018	0.805	0.981	0.820
let-7a-5p	1829–1850	0.835	3′UTR^h^	−21.500	−12.736	0.631	0.001	0.357
let-7a-5p	1,161–1,177	0.831	CDS	−20.100	−19.630	0.767	0.900	0.816
let-7a-5p	1,161–1,179	0.808	CDS	−22.300	−21.904	0.783	0.910	0.816
miR-205-5p	1,025–1,031	0.787	CDS	−17.500	−10.665	0.602	0.997	0.701
miR-22-3p	534–570	0.762	CDS^i^	−21.100	2.9490	0.448	0.963	0.285
miR-31-5p	1,158–1,177	0.755	CDS	−21.800	-16.974	0.667	0.912	0.813
miR-205-5p	835–848	0.755	CDS	−23.700	-13.153	0.442	0.907	0.540
miR-223-3p	859–870	0.727	CDS	−16.200	-6.7460	0.421	0.803	0.560
**Binding regions in RB1**								
let-7a-5p	2,506–2,517	0.953	CDS^i^	−18.100	−16.708	0.823	0.999	0.840
miR-130a-3p	2,529–2,537	0.917	CDS	−17.100	−11.446	0.708	1.000	0.848
miR-31-5p	4,704–4,721	0.912	3′UTR	−20.100	−17.244	0.651	1.000	0.963
let-7a-5p	4,686–4,712	0.911	3′UTR	−22.100	−17.127	0.608	0.999	0.953
miR-31-5p	4,704–4,719	0.899	3′UTR	−18.700	−16.076	0.627	1.000	0.963
miR-31-5p	4,704–4,717	0.898	3′UTR	−17.200	−14.545	0.672	1.000	0.963
let-7a-5p	4,686–4,719	0.896	3′UTR	−20.700	−14.420	0.573	1.000	0.953
miR-130a-3p	2,529–2,534	0.892	CDS	−15.900	−10.778	0.646	1.000	0.848
miR-142-3p	2002–2026	0.883	CDS	−18.500	−12.601	0.692	0.847	0.659
let-7a-5p	562–571	0.878	CDS	−15.500	−14.118	0.846	0.957	0.142

a Start and end position of the target region (site) predicted to be bound by miRNA.

b Probability of the site being an miRNA binding site as predicted by our nonlinear logistic model.

c A measure of stability for miRNA:target hybrid as computed by RNAhybrid.

d A measure of the total energy change of the hybridization.

e A measure of structural accessibility as computed by the average probability of a nucleotide being single-stranded (i.e., unpaired) for the nucleotides in the predicted binding site.

f Conservation score by the PhastCons program for the binding site.

g Relative starting location of the predicted binding site along the length of the sequence (e.g., for 3ʹ′ UTR, 0 indicates the 5ʹ′ end of the UTR, and one corresponds to the 3ʹ′ end).

h “seed” region in 3′UTR (“seed” position: 1843–1849, binding: 8mer).

i “seed” region in CDS (“seed” position: 564–570, binding: 8mer).

Figure 2 Top 10 miRNAs binding site regions identified in the *TP53* gene.

**FIGURE 3:**
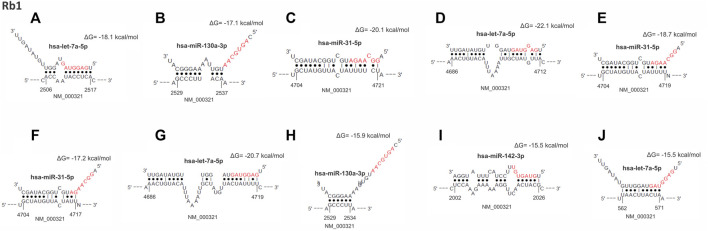
From **(A–J)**, hybrid mRNA (RB1)—miRNA upregulated. The upper and lower sequences represent the miRNA in the 3′-5′ sense and 5′-3′ sense hybridization sites in the mRNA, respectively. The numbers at the ends of the mRNA show the starting and ending position of the hybridization sites. Dots between the sequences indicate the paired nucleotides. Nucleotides in red mark the “seed” sequence in miRNA. The energy resulting from the hybridization was calculated by the RNA*hybrid* algorithm.

The lower expression of *RB1* gene was also found in 69% of the tumors. Interestingly, we observed that the thirteen overexpressed miRNAs that down-regulated *TP53* also regulated *RB1* expression (Figure 3). A total of 490 miRNA binding sites were identified for *RB1* (Supplementary Table S3), of which 477 (97.3%) were located in the non-canonical seedless regions, while 13 (2.7%) were in the seed regions. Bindings in the seed regions occurred with seven overexpressed miRNAs (miR-93-5p, let-7a-5p, miR-25-3p, miR-130a-3p, miR-200c-3p, miR-205-5p, and miR-142-3p), most of which were 7mer-A1 (46.2%). Other binding sites identified in the seed regions were offset-6mer (23.0%), 6mer, and 7mer-m8 (15.4%, each) (Supplementary Table S2A). The *RB1* gene also presented the highest number of miRNA target sites in seedless regions (490 in total), in which all 13 differentially expressed miRNAs can bind. The *RB1* coding region also had the highest number of seedless regions (57.0%), followed by 3′UTR (42.4%) and 5′UTR (0.6%). The miRNA let-7a-5p showed the highest number of seedless bindings (77 predicted sites), followed by miR-93-5p (74 predicted sites). The miRNAs presenting a smaller number of regions were miR-16-5p, miR-205-5p, miR-223-3p (22 predicted sites, each) and miR-22-3p (19 predicted sites). The *RB1* gene also presented hotspots regions where several miRNAs can bind. The intervals between 2,202–2297pb and 1906–1997 pb house a total of 21, and 19 sites, respectively (NCBI Reference Sequence: NM_000321.3). Table 3 shows the top 10 binding sites in *RB1*.

Figure 3 Top 10 miRNAs binding site regions identified in the *RB1* gene.

### Molecular Pathways

**FIGURE 4:**
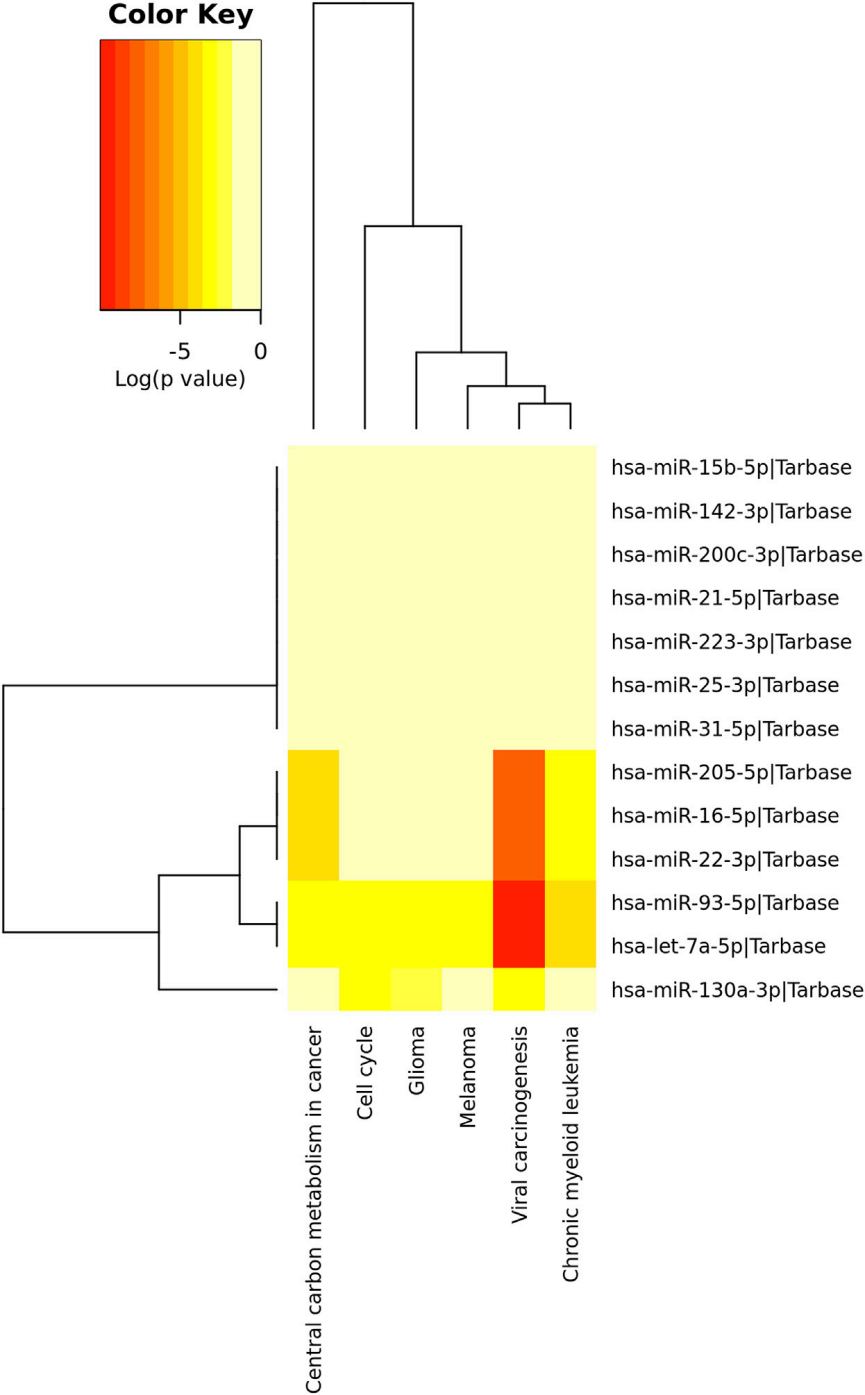
Top pathways identified by the tarbase algorithm (*p* < 0.05; DIANA/miRPath v.3). Red colors indicate a stronger role of the miRNAs on a given pathways as compared to the lighter colors.

KEGG pathway analysis was performed to identify the involvement of the 13 upregulated miRNAs above in disease and signaling pathways. This analysis revealed a total of 13 KEGG pathways (Supplementary Table S3), of which the top was: viral carcinogenesis (hsa05203) (*p*=<1.00 × 10^−325^), central carbon metabolism in cancer (hsa05230) (*p* = 3.39 × 10^−06^), chronic myeloid leukemia (hsa05220) (*p* = 1.33 × 10^−05^), glioma (hsa05214) (*p* = 0.0064), melanoma (hsa05218) (*p* = 0.0120) and cell cycle (hsa04110) (*p* = 0.0224) (Figure 4; Table 4).

**TABLE 4:** Top six molecular pathways involving overexpressed miRNAs targeting *TP53* and *RB1* genes.

KEGG Pathway	*p-*Value pathway	miRNAs name	Target gene	*p-*Value interaction
Viral carcinogenesis (hsa05203)	<1.00 × 10^−325^	let-7a-5p	*TP53* and *RB1*	1.16 × 10^−10^
		miR-130a-3p	*RB1*	0.0039733
		miR-16-5p	*TP53*	6.44 × 10^−08^
		miR-205-5p	*TP53*	6.44 × 10^−08^
		miR-22-3p	*TP53*	6.44 × 10^−08^
		miR-93-5p	*TP53* and *RB1*	1.16 × 10^−10^
Central carbon metabolism in cancer (hsa05230)	3.39 × 10^−06^	let-7a-5p	*TP53*	0.0011443
		miR-16-5p	*TP53*	0.0005629
		miR-205-5p	*TP53*	0.0005629
		miR-22-3p	*TP53*	0.0005629
		miR-93-5p	*TP53*	0.0011443
Chronic myeloid leukemia (hsa05220)	1.33 × 10^−05^	let-7a-5p	*TP53* and *RB1*	0.0005998
		miR-16-5p	*TP53*	0.0041602
		miR-205-5p	*TP53*	0.0041602
		miR-22-3p	*TP53*	0.0041602
		miR-93-5p	*TP53* and *RB1*	0.0005998
Glioma (hsa05214)	0.0064303	let-7a-5p	*TP53* and *RB1*	0.0017756
		miR-130a-3	*RB1*	0.0105785
		miR-93-5p	*TP53* and *RB1*	0.0017756
Melanoma (hsa05218)	0.0120334	let-7a-5p	*TP53* and *RB1*	0.0041201
		miR-93-5p	*TP53* and *RB1*	0.0041201
Cell cycle (hsa04110)	0.0224147	let-7a-5p	*TP53* and *RB1*	0.0038093
		miR-130a-3	*RB1*	0.0014269
		miR-93-5p	*TP53* and *RB1*	0.0038093

Figure 4 Unsupervised hierarchical grouping of the 13 miRNAs differentially expressed and top related pathways.

## Discussion

It is well known that the integration of the human papillomavirus (HPV) can occur at or near cancer-related genes (Durst et al., 1987). However, it is not completely understood the mechanisms by which the HPV virus controls its integration into the host cell genome and the molecular consequences that ultimately lead to the development and progression of the HPV infected tumors. Studies have used high-performance technologies to identify virus integration sites in the host genome to better understand the molecular alterations that occur in the host cell, leading to the loss of its genomic stability (Akagi et al., 2014; Bodelon et al., 2016; Liu et al., 2016; Gao et al., 2017; Rosa et al., 2019). The most well-known example is the canonical HPV/*TP53*/*RB1* signaling pathway initiated by the viral E2 disruption. This leads to the loss of the negative feedback control of the viral oncoproteins E6 and E7, mediating ubiquitination and degradation of the p53 and pRb proteins, respectively (Squarzanti et al., 2018). Other authors proposed that HPV integration also directly causes activation of oncogenes or inactivation of tumor suppressors, as reported in HPV-related squamous cell carcinomas (Parfenov et al., 2014; Hu et al., 2015).

In our previous study, we showed downregulated mRNA expression of the *TP53* and *RB1* genes in 86 and 65% of high-risk HPV-associated PeCa, respectively. In the present subset of cases, we evaluated miRNAs’ expression, and observed that 73 and 69% were downregulated for both genes, respectively, suggesting the existence of other regulatory mechanisms in addition to the canonical HPV/*TP53*/*RB1* pathway (Macedo et al., 2020). Although not all the cases presented with expression alterations in these genes, these results were recently corroborated by Furuya *et al.*, who also described *TP53* reduced expression levels in penile tumors.

Compared to other cancers (Santos et al., 2018; Datta et al., 2019; Hussen et al., 2021; Liu et al., 2021), few studies have described epigenetic events in penile tumors, whether evaluating miRNAs (Zhang et al., 2015; Hartz et al., 2016; Kuasne et al., 2017; Peta et al., 2017; Ayoubian et al., 2021; Furuya et al., 2021) or by evaluating methylation patterns (Feber et al., 2015; Kuasne et al., 2015; Marchi et al., 2017). Changes by both mechanisms could justify the downregulation of *TP53* and *RB1*, however only *RB1* has been reported to be hypermethylated (Marchi et al., 2017). Additionally, as most pathogenic variants of these genes have been described in coding regions, the mRNA downregulation of *TP53* and *RB1* does not appear to be due to mutations (Feber et al., 2016; Wang et al., 2019; Chahoud et al., 2021). Furthermore, few studies have evaluated patients’ cohorts with a high incidence of HPV infection, remaining poorly known the impact of HPV infection in disrupting mRNA/miRNA networks in penile tumors (Zhang et al., 2015; Hartz et al., 2016; Kuasne et al., 2017; Ayoubian et al., 2021; Furuya et al., 2021). In the present study, our main goal was to determine whether altered miRNAs expression could be associated with the down-regulation of the *TP53* and *RB1* expression in the etiopathogenesis of HPV-associated PeCa. This goal is of critical relevance to these particular virus associated with infected tumors, considering that the patient cohort investigated, from the State of Maranhão in Northeastern Brazil, is characterized by advanced PeCa and a high rate of HPV infection (>90%), as we reported previously (Coelho et al., 2018; Macedo et al., 2020). In addition, this study can provide useful information to target HPV-specific molecular pathways in human cancers.

In the present study, all patients were tested for HPV by nested-PCR followed by DNA sequencing. Using two highly sensitive methods we successfully detected HPV infection in 100% of men with PeCa, all of them with high-risk subtypes. Despite the high HPV prevalence in all human populations, occurring as hundreds of types, subtypes, and variants, many of them are not associated with cancer. On the other hand, it is well established the correlation between high-risk HPV and severe dysplasia, *in situ* and invasive cancer, usually as monoclonal lesions due to clonal selection from less advanced precursors (Pontén and Guo, 1998). This may explain why we were able to detect HPV in 100% of the primary tumor since all of them presented high-risk genotypes.

It is well established that p53 regulates the expression of both protein-coding genes and non-coding RNAs (Hermeking, 2012; Fischer, 2017). *TP53*-regulated miRNAs can mediate tumor suppression in response to cellular stress; similarly, the expression and activity of p53 can also be under the control of miRNAs (Hermeking, 2012). More than 20 miRNAs have been described to directly regulate p53 via canonical bindings (seed) in 3′UTR (reviewed by Liu et al., 2017). Down regulation of *TP53* through seed sequences induce phenotypes that are consistent with loss of p53 function, such as reduced apoptosis, cellular senescence, increased invasion, and growth of tumor cells (Hermeking, 2012; Deng and Sui, 2013; Hermeking et al., 2014). Despite the increasing number of miRNAs that form the TP53 mRNA/miRNAs interaction network, there is no information on *TP53*-repressor miRNAs in HPV-associated PeCa. Our data revealed a total of 507 differentially expressed miRNA (miRDE) between the tumor and non-tumor tissue of HPV-infected PeCa patients, of which 494 were downregulated and 13 were upregulated. Among the 13 miRDE upregulated, five (let-7a-5p, miR-130a-3p, miR-15b-5p, miR-21-5p and miR-25-3p) were found overexpressed in 100% of the tumors analyzed. Moreover, miR-130a-3p, miR-15b-5p and miR-31-5p were predicted as novel regulator for *TP53* gene; while miR-142-3p, miR-200c-3p, miR-205-5p, miR-223-3p, miR-22-3p, miR-25-3p and miR-31-5p for *RB1*.

Several studies have shown up-regulated expression of these miRNAs in several types of tumors. Overexpression of let-7a-5p has been observed in HCV-related cirrhosis (Petkevich et al., 2021) and liver cancer and ovarian cancer, where it presents a non-invasive diagnostic potential (Liu et al., 2021). Corroborating our data, some studies have also suggested that *TP53* is a target of let-7a-5p (Balakrishnan et al., 2014; Pillai et al., 2014; Nunez Lopez et al., 2019; Zhou et al., 2019). MiR-130a-3p is recognized as a miRNA with tumor suppressor action (Kong et al., 2018; Song et al., 2021), that may act directly (Causin et al., 2021) or indirectly (Hu et al., 2021) in cancer progression. On the other hand, miR-15b-5p has generally been described to act on cell proliferation mechanisms, such as the ones involving the *LATS2* (Liu et al., 2020), *BCL-2* (Zhang et al., 2015)*,* and *PTPN4*/*STAT3* pathways (Liu et al., 2020).

Interestingly, down-regulation of *TP53* by miR-25 resulted in a decrease in apoptosis in HCT116 colon cancer cells, A549 cells, NSCLC, and multiple myeloma cells (Kumar et al., 2011). In lung cancer, miR-25 was observed to promote cell proliferation and also inhibit apoptosis by down-regulating the expression of the *MOAP1*/*TP53* axis genes (Wu et al., 2015). Moreover, recent evidence shows that miR-25-3p may also act with LncRNAs on a *LINC00858*/miR-25/*SMAD7* axis modulating *TP53*-wild expression in colorectal carcinoma (Zhan et al., 2020). (Wang et al., 2021) also observed that exosomal miR-25-3p induced cell proliferation and resistance to temozolomide in glioblastoma through down-regulation of *FBXW7*, promoting c-Myc and cyclin E expression. MiR-21-5p, also observed up-regulated in this study, was shown by (Huang et al., 2021) to negatively regulate the tumor suppressor *PDCD4* and cause resistance to by Osimertinib by interfering with MEK/ERK signaling.

These results are in concordance with the suppressive effect of these miRNAs in the HPV-related genes observed in PeCa in the present study. Our computational analysis revealed that *TP53* and *RB1* have 131 and 490 target sites for the 13 upregulated miRNAs, respectively. The highest number of miRNA binding sites were identified in coding regions, and not in UTR regions, as reported by (Hafner et al., 2010) Furthermore, 98.5 and 97.3% of the sites in *TP53* and *RB1*, respectively, are in non-canonical seedless regions, presenting high levels of complementarity and conservation. Although most miRNA targets have sites that are perfectly complementary to the seed region, it has been shown that miRNAs can directly interact with seedless binding sequences, even improving their function (Shin et al., 2010). (Lal et al., 2009) for example, presented evidence of cell proliferation control by miR-24 in the *E2F2*/*MYC* axis through seedless binding in the 3′UTR region. (Park et al., 2017). also showed that destabilization of miRNAs targets is dramatically increased when binding occurs in non-canonical seedless regions. Therefore, we propose that the 13 miRNAs overexpressed in PeCa directly repress *TP53* and *RB1* by silencing their messenger RNA at different binding sites, especially in non-canonical seedless regions.

Although a unique miRNA may have a pivotal role in a particular pathway, most miRNAs act targeting multiple mRNAs, affecting the same or several gene pathways. Considering the 13 upregulated miRNAs, we predicted six main pathways by the enrichment analysis. Viral carcinogenesis (hsa05203), in which *TP53* and *RB1* act, was the main pathway affected (*p* < 1.00 × 10-325). MiR-205-5p, miR-16-5p, miR-22-3p, miR-93-5p, let-7a-5p, and miR-130a-3p were involved in most of the pathways affected. Glioma and cell cycle pathways, in addition to viral carcinogenesis, were previously shown to be regulated by other miRNAs identified in cytobands affected by CNVs in the same population from Maranhão State (Silva et al., 2021). Thus, these current findings reinforce the involvement of these pathways in HPV-associated penile tumorigenesis.

It is worth highlighting that 84.6% of miRDE were observed to be located in HPV integration sites (HPV-IS), including the five miRDE overexpressed in 100% of cases (sites at 3q25.33, 7q22.1, 9q22.32, 11q12.1, and 17q23.1). Several viruses mediate tumorigenesis by expressing viral oncogenes or activating host oncogenes through the integration of viral DNA into the human genome (Lee and Dutta, 2009; Tuna and Amos, 2017). We have recently shown that chromosomal regions with gene copy number alterations (CNA) are present in HPV-IS, such as 2p12-p11.2 and 14q32.33 (observed in 100% of PeCa patients), which can also affect the expression of miRNAs located in these regions (Macedo et al., 2020; Silva et al., 2021). These regions were also described in other HPV-associated tumors (Wentzensen et al., 2004; Kumar Gupta and Kumar, 2015; Holmes et al., 2016; Liu et al., 2016). This data shows the close connection of CNAs and miRNA deregulation located in HPV-IS. Altogether, our present data, support that miRNAs located in HPV-IS can directly repress genes related to HPV infection, such as *TP53* and *RB1*, highlighting HPV insertion as one of the factors that trigger epigenetic mechanisms.

## Conclusion

In this study, we suggest that the HPV-related genes, *TP53* and *RB1*, are directly down-regulated by 13 miRNAs located in high-risk HPV integration sites, notably for the HPV16 subtype, present in 72% of the PeCa patients studied. Considering that the expression and activity of *TP53* and *RB1* can be under the control of miRNAs, our findings provide a new understanding of the role of high-risk HPV infection in penile tumorigenesis through an epigenetic mechanism.

## Data Availability

The datasets presented in this study can be found in online repositories. The names of the repository/repositories and accession number(s) can be found below: https://www.ncbi.nlm.nih.gov/geo/, GSE197121.
