# Supplementary material for: Upregulated miRNAs on the TP53 and RB1 Binding Seedless Regions in High-Risk HPV-Associated Penile Cancer
Source: Front Genet. 2022 Jun 24;13:875939. doi: 10.3389/fgene.2022.875939 (PMC9263206; doi:10.3389/fgene.2022.875939)
Supplement: Supplementary file 1 [file DataSheet1.PDF]

Supplementary Material

1 Supplementary Tables

Supplementary Table S1. Detailed clinical-histopathological profile and social habits of patients diagnosed with penile carcinoma enrolled in this study (n =22).

Case	Age	Phymosis	Smoking	Alcoholism	HPV	Tumor site	Histological subtype	Predominant lesion	Penectomy	Tu. Size	Tu. Grade	Tu. Stage	Lymphatic invasion	Perineural invasion	*TP53* expression	*RB1* expression
T01	62	no	N/A	N/A	16	glans	keratinized penile carcinoma	ulcerated	total	2.5	II	pT3	yes	yes	downregulated	downregulated
T02	80	N/A	smoker former	N/A	16	glans, foreskin and other areas	condilomatous	ulcerated	total	10	II	pT2	no	no	downregulated	downregulated
T03	81	yes	yes	N/A	18,53	glans and foreskin	condilomatous	ulcerated	total	2.1	II	pT1	no	no	downregulated	downregulated
T04	37	yes	no	yes	positive	glans and foreskin	keratinized penile carcinoma	nodular ulcerated	total	5	III	pT3	no	yes	N/A	N/A
T05	76	yes	no	yes	30	glans and foreskin	condilomatous	ulcerated	partial	3	I	pT1b	yes	no	downregulated	downregulated
T06	54	yes	yes	yes	positive	foreskin	keratinized penile carcinoma	vegetative	partial	0.8	II	pT2	yes	no	N/A	N/A
T07	74	yes	no	no	73,74	glans, foreskin and other areas	keratinized and basaloid	verrucosa	partial	0.8	I	pT2	no	no	downregulated	downregulated
T08	78	N/A	yes	yes	16,06	glans	keratinized and basaloid	vegetative	partial	4	III	pT1b	no	no	N/A	downregulated
T09	44	yes	no	yes	16	glans, foreskin and other areas	keratinized penile carcinoma	ulcerated	partial	1.7	II	pT1	no	no	downregulated	N/A
T10	57	no	no	yes	58	glans	keratinized and condilomatous	nodular ulcerated	total	3	III	pT2	no	no	N/A	N/A
T11	85	no	N/A	N/A	16	glans and foreskin	condilomatous	nodular ulcerated	partial	4	III	pT3	no	yes	N/A	upregulated

T12	83	no	smoker former	alcoholic former	16	glans and foreskin	keratinized penile carcinoma	ulcerated	partial	4.5	III	pT3	no	yes	Normal	upregulated
T13	40	no	no	no	59,66	glans	condilomatous	ulcer-vegetative	partial	3	II	pT2	no	no	downregulated	upregulated
T14	68	N/A	no	no	16,66	glans and foreskin	condilomatous	verrucosa	partial	5.5	I	pT3	no	no	upregulated	upregulated
T15	65	N/A	N/A	N/A	16,74	glans and foreskin	condilomatous	vegetative	partial	3.5	II	pT2	no	no	downregulated	downregulated
T16	51	N/A	yes	yes	16,35,59	glans, foreskin and other areas	keratinized penile carcinoma	ulcero-vegetante	partial	3	II	pT2	yes	no	downregulated	N/A
T17	74	no	yes	no	positive	glans	condilomatous	ulcerated	partial	1.8	II	pT1a	no	no	downregulated	N/A
T18	32	no	yes	yes	16,44,74	glans	keratinized and condilomatous	ulcer-vegetative	partial	3.7	II	pT2	no	no	N/A	downregulated
T19	71	no	no	no	16	glans	keratinized penile carcinoma	ulcerated	partial	3.2	III	pT3	no	no	downregulated	N/A
T20	69	no	no	no	16,30	glans	condilomatous	vegetative	partial	3.5	I	pT2	no	no	N/A	downregulated
T21	73	yes	smoker former	no	16	foreskin	keratinized penile carcinoma	ulcerated	postectomy	N/A	II	pT1	N/A	N/A	downregulated	N/A
T22	59	yes	no	yes	positive	glans	condilomatous	verrucous	partial	5.5	II	pT3	no	yes	N/A	N/A

N/A = not available; a = No information on HPV genotype; Tu = tumor (mm); Genotyping was performed by nested-PCR and DNA sequencing, except for T04, T06, T17 and T22 because did not have enough DNA for both assays.

Supplementary Table S2A. miRNA binding *'seed'* regions identified in *TP53* and *RB1* genes.

miRNA	Gene	Site Position*	LogitProb[a]	Region	Seed Position[b]	Seed Type[c]	ΔG_{hybrid}[d]	ΔG_{total}[e]	Site Access[f]	Seed Access[g]	Site Consv[h]	Seed Consv[i]	Site Location[j]
hsa-miR-22-3p	*TP53*	534-570	0.762	CDS	564-570	8mer	-21.100	2.949	0.448	0.273	0.963	0.993	0.285
hsa-let-7a-5p	*TP53*	1829-1850	0.835	3'UTR	1843-1849	8mer	-21.500	-12.736	0.631	0.627	0.001	0	0.357
hsa-let-7a-5p	*RB1*	2506-2517	0.953	CDS	2511-2516	7mer-A1	-18.100	-16.708	0.823	0.886	0.999	1	0.840
hsa-let-7a-5p	*RB1*	2085-2103	0.827	CDS	2097-2103	7mer-m8	-19.900	-12.574	0.619	0.968	0.817	1	0.689
hsa-let-7a-5p	*RB1*	2915-2935	0.805	CDS	2929-2934	7mer-A1	-19.700	-12.982	0.524	0.431	0.965	1.000	0.986
hsa-miR-130a-3p	*RB1*	633-648	0.799	CDS	642-648	7mer-m8	-15.900	-12.886	0.566	0.296	0.994	1.000	0.168
hsa-miR-200c-3p	*RB1*	760-784	0.787	CDS	778-783	7mer-A1	-18.200	-6.342	0.474	0.411	0.999	1	0.213
hsa-miR-205-5p	*RB1*	2793-2809	0.787	CDS	2803-2808	7mer-A1	-19.200	-9.408	0.478	0.476	0.980	0.994	0.943
hsa-miR-205-5p	*RB1*	1402-1428	0.768	CDS	1422-1427	7mer-A1	-21.200	-1.732	0.491	0.335	0.975	0.960	0.443
hsa-miR-93-5p	*RB1*	4333-4353	0.765	3'UTR	4347-4352	6mer	-19.100	-12.903	0.532	0.625	0.990	0.998	0.759
hsa-let-7a-5p	*RB1*	3612-3645	0.703	3'UTR	3639-3644	7mer-A1	-20.200	-0.817	0.428	0.178	0.969	1	0.362
hsa-miR-142-3p	*RB1*	1285-1311	0.667	CDS	1305-1310	offset-6mer	-21.400	-7.625	0.479	0.488	0.999	1.000	0.402
hsa-let-7a-5p	*RB1*	2477-2502	0.662	CDS	2496-2501	offset-6mer	-20.000	-4.112	0.472	0.743	0.968	0.996	0.829
hsa-miR-25-3p	*RB1*	4167-4185	0.604	3'UTR	4180-4185	offset-6mer	-17.000	-7.796	0.415	0.291	0.429	0.812	0.667
hsa-miR-25-3p	*RB1*	3381-3416	0.559	3'UTR	3411-3416	6mer	-13.600	-0.088	0.574	0.646	0.719	0.999	0.235

* Start and end position of the target region (site) predicted to be bound by miRNA; [a] Probability of the site being an miRNA binding site as predicted by our nonlinear logistic model; [b] start and end position of the target sub-region complementary to the miRNA seed (i.e. positions 2-7/8 of the miRNA); [c] 6mer, offset 6mer, 7mer-A1, 7mer-m8, and 8mer seed sites; [d] A measure of stability for miRNA:target hybrid as computed by RNAhybrid; [e] A measure of the total energy change of the hybridization; [f] A measure of structural accessibility as computed by the average probability of a nucleotide being single-stranded (i.e., unpaired) for the nucleotides in the predicted binding site; [g] A measure of structural accessibility as computed by the average of single-stranded probabilities of the nucleotides in the target sub-region complementary to the miRNA seed; [h] Conservation score by the PhastCons program for the binding site; [i] Conservation score by the PhastCons program for the target sub-region complementary to the miRNA seed and [j] Relative starting location of the predicted binding site along the length of the sequence (e.g., for 3″ UTR, 0 indicates the 5″ end of the UTR, and 1 corresponds to the 3″ end).

Supplementary Table S2B. miRNA binding *'seedless'* regions identified in *TP53* gene.

miRNA	Gene	Site Position*	LogitProb[a]	Region	ΔG_{hybrid}[b]	ΔG_{total}[c]	Site Access[d]	Site Consv[e]	Site Location[f]
hsa-miR-205-5p	*TP53*	835-845	0.666	CDS	-20.700	-10.990	0.502	0.899	0.540
hsa-miR-130a-3p	*TP53*	1147-1175	0.546	CDS	-22.600	-3.107	0.511	0.788	0.804
hsa-miR-142-3p	*TP53*	835-851	0.576	CDS	-17	-6.646	0.515	0.923	0.540
hsa-miR-223-3p	*TP53*	920-935	0.538	CDS	-19.300	-5.720	0.521	0.938	0.612
hsa-miR-130a-3p	*TP53*	635-645	0.508	CDS	-17.500	-9.863	0.525	0.250	0.371
hsa-let-7a-5p	*TP53*	1340-1359	0.506	CDS	-15.600	-1.373	0.526	0.479	0.967
hsa-miR-16-5p	*TP53*	1057-1073	0.533	CDS	-17.800	-6.503	0.533	0.931	0.728
hsa-let-7a-5p	*TP53*	1026-1046	0.514	CDS	-15.300	3.662	0.534	0.820	0.701
hsa-miR-205-5p	*TP53*	1025-1040	0.688	CDS	-24.500	-12.477	0.537	0.997	0.701
hsa-miR-22-3p	*TP53*	1012-1026	0.530	CDS	-16	-4.737	0.537	0.742	0.690
hsa-miR-142-3p	*TP53*	1009-1032	0.519	CDS	-15.100	-1.908	0.538	0.838	0.687
hsa-miR-21-5p	*TP53*	1010-1023	0.635	CDS	-15.200	-3.538	0.542	0.723	0.688
hsa-let-7a-5p	*TP53*	312-330	0.628	CDS	-19.200	-10.285	0.548	0.002	0.097
hsa-miR-15b-5p	*TP53*	1138-1149	0.501	CDS	-18.100	-11.028	0.558	0.524	0.796
hsa-miR-200c-3p	*TP53*	1009-1023	0.707	CDS	-21.800	-10.288	0.559	0.742	0.687
hsa-miR-142-3p	*TP53*	1152-1176	0.629	CDS	-21.200	-6.168	0.562	0.927	0.808
hsa-miR-22-3p	*TP53*	1012-1029	0.520	CDS	-16.800	-5.746	0.564	0.785	0.690
hsa-miR-21-5p	*TP53*	1010-1026	0.635	CDS	-15.900	-4.153	0.575	0.772	0.688
hsa-miR-25-3p	*TP53*	309-315	0.664	CDS	-16.200	-9.594	0.581	0.000	0.095
hsa-miR-200c-3p	*TP53*	1009-1026	0.716	CDS	-23.100	-11.503	0.587	0.785	0.687
hsa-miR-21-5p	*TP53*	1010-1029	0.637	CDS	-16.600	-5.061	0.593	0.806	0.688
hsa-miR-205-5p	*TP53*	1025-1031	0.787	CDS	-17.500	-10.665	0.602	0.997	0.701
hsa-let-7a-5p	*TP53*	312-328	0.681	CDS	-15.700	-10.053	0.610	0.002	0.097
hsa-let-7a-5p	*TP53*	1180-1195	0.611	CDS	-18.800	-9.278	0.611	0.884	0.832
hsa-miR-200c-3p	*TP53*	1009-1021	0.593	CDS	-16.100	-5.376	0.642	0.702	0.687
hsa-miR-21-5p	*TP53*	1255-1260	0.501	CDS	-11.200	-5.966	0.663	0.457	0.895
hsa-miR-31-5p	*TP53*	1158-1177	0.755	CDS	-21.800	-16.974	0.667	0.912	0.813

hsa-miR-93-5p	*TP53*	1166-1174	0.864	CDS	-15.900	-15.316	0.714	0.959	0.820
hsa-let-7a-5p	*TP53*	1161-1177	0.831	CDS	-20.100	-19.630	0.767	0.900	0.816
hsa-miR-200c-3p	*TP53*	1009-1018	0.715	CDS	-15.900	-11.206	0.777	0.618	0.687
hsa-let-7a-5p	*TP53*	1161-1179	0.808	CDS	-22.300	-21.904	0.783	0.910	0.816
hsa-miR-93-5p	*TP53*	1166-1186	0.848	CDS	-18.400	-18.018	0.805	0.981	0.820
hsa-miR-200c-3p	*TP53*	1177-1190	0.566	CDS	-15	-8.087	0.823	0.998	0.829
hsa-miR-200c-3p	*TP53*	384-396	0.617	CDS	-17.500	-6.698	0.402	0.000	0.158
hsa-miR-205-5p	*TP53*	1025-1036	0.690	CDS	-24.200	-11.901	0.403	0.996	0.701
hsa-miR-130a-3p	*TP53*	672-682	0.535	CDS	-19.800	-10.837	0.404	0.998	0.402
hsa-miR-205-5p	*TP53*	390-404	0.501	CDS	-15.600	-4.991	0.404	0	0.163
hsa-let-7a-5p	*TP53*	617-642	0.581	CDS	-22.100	-6.195	0.407	0.593	0.355
hsa-miR-93-5p	*TP53*	850-866	0.695	CDS	-17.100	-7.102	0.410	0.864	0.552
hsa-miR-93-5p	*TP53*	1039-1055	0.519	CDS	-20.800	-4.274	0.411	0.608	0.712
hsa-miR-16-5p	*TP53*	840-864	0.642	CDS	-18.700	-0.741	0.413	0.931	0.544
hsa-miR-15b-5p	*TP53*	840-864	0.592	CDS	-16.900	1.059	0.413	0.931	0.544
hsa-let-7a-5p	*TP53*	903-922	0.504	CDS	-16.900	3.803	0.417	1.000	0.597
hsa-miR-31-5p	*TP53*	606-616	0.535	CDS	-15.400	-6.172	0.418	0.990	0.346
hsa-miR-200c-3p	*TP53*	384-400	0.532	CDS	-17.600	-3.543	0.419	0.000	0.158
hsa-miR-223-3p	*TP53*	859-870	0.727	CDS	-16.200	-6.746	0.421	0.803	0.560
hsa-miR-205-5p	*TP53*	1025-1034	0.634	CDS	-18.400	-5.023	0.422	0.996	0.701
hsa-miR-200c-3p	*TP53*	548-568	0.540	CDS	-20.800	-2.929	0.423	0.994	0.297
hsa-miR-205-5p	*TP53*	835-863	0.632	CDS	-22.800	-2.410	0.424	0.911	0.540
hsa-miR-93-5p	*TP53*	707-727	0.511	CDS	-21.100	-2.739	0.430	0.880	0.431
hsa-miR-93-5p	*TP53*	1133-1149	0.627	CDS	-22.600	-15.369	0.433	0.530	0.792
hsa-miR-16-5p	*TP53*	1133-1149	0.511	CDS	-21.100	-13.869	0.433	0.530	0.792
hsa-miR-223-3p	*TP53*	1409-1424	0.642	CDS	-16.500	-9.699	0.436	0.049	1.025
hsa-miR-93-5p	*TP53*	557-572	0.548	CDS	-21.600	-7.769	0.436	0.993	0.305
hsa-miR-93-5p	*TP53*	850-868	0.674	CDS	-19.300	-8.099	0.437	0.878	0.552
hsa-miR-200c-3p	*TP53*	384-402	0.548	CDS	-20	-4.954	0.437	0.000	0.158
hsa-miR-25-3p	*TP53*	1015-1038	0.551	CDS	-20	-6.818	0.438	0.904	0.692
hsa-miR-16-5p	*TP53*	840-859	0.631	CDS	-18.100	-2.110	0.439	0.969	0.544
hsa-miR-205-5p	*TP53*	835-848	0.755	CDS	-23.700	-13.153	0.442	0.907	0.540

hsa-miR-142-3p	*TP53*	835-848	0.617	CDS	-15.400	-4.853	0.442	0.907	0.540
hsa-miR-93-5p	*TP53*	1133-1146	0.568	CDS	-19.300	-12.494	0.446	0.637	0.792
hsa-miR-93-5p	*TP53*	1354-1379	0.542	CDS	-15.800	-0.012	0.449	0.693	0.979
hsa-miR-21-5p	*TP53*	887-899	0.548	CDS	-15.100	-2.312	0.450	1	0.584
hsa-miR-93-5p	*TP53*	956-971	0.532	CDS	-16.200	-6.461	0.455	0.952	0.642
hsa-miR-93-5p	*TP53*	1039-1062	0.572	CDS	-26.200	-9.048	0.462	0.702	0.712
hsa-miR-142-3p	*TP53*	835-856	0.577	CDS	-19.500	-2.881	0.463	0.938	0.540
hsa-let-7a-5p	*TP53*	626-640	0.564	CDS	-16.400	-6.098	0.466	0.527	0.363
hsa-let-7a-5p	*TP53*	1409-1427	0.627	CDS	-17.100	-9.534	0.474	0.042	1.025
hsa-let-7a-5p	*TP53*	626-638	0.555	CDS	-15	-4.709	0.477	0.608	0.363
hsa-miR-200c-3p	*TP53*	1009-1035	0.632	CDS	-24.400	-10.523	0.479	0.855	0.687
hsa-miR-25-3p	*TP53*	309-321	0.625	CDS	-17.200	-9.396	0.479	0.000	0.095
hsa-miR-205-5p	*TP53*	835-843	0.642	CDS	-16.400	-8.957	0.482	0.888	0.540
hsa-miR-205-5p	*TP53*	310-322	0.579	CDS	-17.100	-8.965	0.482	0.000	0.096
hsa-miR-130a-3p	*TP53*	1401-1422	0.516	CDS	-16.900	-8.458	0.482	0.036	1.019
hsa-miR-93-5p	*TP53*	1354-1377	0.506	CDS	-15.200	3.774	0.484	0.676	0.979
hsa-miR-15b-5p	*TP53*	553-571	0.500	CDS	-17.500	-3.685	0.484	0.994	0.301
hsa-miR-22-3p	*TP53*	1012-1023	0.543	CDS	-15	-3.822	0.489	0.677	0.690
hsa-miR-205-5p	*TP53*	1025-1049	0.515	CDS	-28.600	-2.910	0.489	0.732	0.701
hsa-miR-205-5p	*TP53*	1397-1418	0.534	CDS	-17.600	-10.818	0.490	0.029	1.015
hsa-miR-93-5p	*TP53*	353-368	0.528	CDS	-17.800	-7.914	0.492	0.027	0.132
hsa-miR-200c-3p	*TP53*	384-393	0.613	CDS	-16.900	-6.214	0.498	4,00E-04	0.158
hsa-miR-200c-3p	*TP53*	1900-1917	0.564	3'UTR	-16.600	-7.381	0.808	0.002	0.413
hsa-let-7a-5p	*TP53*	1787-1807	0.644	3'UTR	-15.300	-11.395	0.749	0.438	0.324
hsa-miR-93-5p	*TP53*	1902-1929	0.508	3'UTR	-15.700	-1.373	0.732	0.001	0.415
hsa-let-7a-5p	*TP53*	1829-1844	0.639	3'UTR	-16.400	-12.640	0.696	0.001	0.357
hsa-let-7a-5p	*TP53*	1786-1809	0.648	3'UTR	-21.200	-14.881	0.670	0.384	0.323
hsa-let-7a-5p	*TP53*	1723-1739	0.569	3'UTR	-15.200	-10.208	0.665	0.006	0.273
hsa-miR-130a-3p	*TP53*	2563-2580	0.559	3'UTR	-16	-5.022	0.657	0.019	0.939
hsa-miR-93-5p	*TP53*	1902-1922	0.510	3'UTR	-15.200	-0.872	0.647	0.001	0.415
hsa-miR-21-5p	*TP53*	2502-2521	0.559	3'UTR	-16.200	-6.312	0.622	0.578	0.891

hsa-miR-31-5p	*TP53*	1822-1838	0.535	3'UTR	-20.400	-8.857	0.598	0.001	0.351
hsa-miR-31-5p	*TP53*	1822-1844	0.643	3'UTR	-24.500	-12.954	0.597	0.001	0.351
hsa-miR-31-5p	*TP53*	2524-2545	0.641	3'UTR	-15.600	-6.032	0.596	0.683	0.908
hsa-miR-21-5p	*TP53*	2502-2526	0.566	3'UTR	-20.700	-10.433	0.582	0.643	0.891
hsa-miR-16-5p	*TP53*	2501-2522	0.575	3'UTR	-17.700	-10.176	0.572	0.566	0.890
hsa-miR-22-3p	*TP53*	2505-2526	0.547	3'UTR	-15.300	-5.430	0.564	0.731	0.893
hsa-let-7a-5p	*TP53*	2525-2541	0.559	3'UTR	-15.300	-7.543	0.563	0.603	0.909
hsa-miR-200c-3p	*TP53*	2506-2523	0.611	3'UTR	-15	-8.168	0.562	0.737	0.894
hsa-miR-31-5p	*TP53*	1822-1836	0.513	3'UTR	-18.600	-7.052	0.546	0.001	0.351
hsa-miR-31-5p	*TP53*	1822-1840	0.588	3'UTR	-23.500	-11.956	0.543	0.001	0.351
hsa-miR-130a-3p	*TP53*	2508-2528	0.612	3'UTR	-16.400	-5.907	0.537	0.860	0.895
hsa-miR-130a-3p	*TP53*	2508-2523	0.652	3'UTR	-16.100	-9.215	0.530	0.828	0.895
hsa-miR-130a-3p	*TP53*	2508-2537	0.515	3'UTR	-16.100	-4.489	0.527	0.764	0.895
hsa-miR-93-5p	*TP53*	1843-1851	0.550	3'UTR	-16.200	-6.998	0.488	0	0.368
hsa-miR-93-5p	*TP53*	2519-2539	0.606	3'UTR	-22.300	-10.560	0.474	0.675	0.904
hsa-miR-31-5p	*TP53*	2525-2534	0.615	3'UTR	-15.200	-7.426	0.459	0.869	0.909
hsa-miR-93-5p	*TP53*	2519-2537	0.600	3'UTR	-20.400	-8.907	0.449	0.719	0.904
hsa-miR-31-5p	*TP53*	2140-2161	0.561	3'UTR	-14.100	-7.042	0.443	0.011	0.603
hsa-miR-31-5p	*TP53*	2491-2511	0.507	3'UTR	-21.200	-11.095	0.436	0.127	0.882
hsa-miR-93-5p	*TP53*	2518-2528	0.644	3'UTR	-19.700	-9.443	0.424	0.879	0.903
hsa-miR-223-3p	*TP53*	1675-1690	0.575	3'UTR	-19.900	-4.840	0.418	0.000	0.235
hsa-miR-142-3p	*TP53*	1632-1645	0.522	3'UTR	-20.200	-4.869	0.413	0.004	0.201
hsa-miR-223-3p	*TP53*	1675-1687	0.598	3'UTR	-16.600	-5.940	0.404	0.000	0.235
hsa-miR-205-5p	*TP53*	206-223	0.523	5'UTR	-16.700	4.951	0.557	0.029	1.046
hsa-miR-130a-3p	*TP53*	16-30	0.542	5'UTR	-17.200	-4.262	0.500	0.943	0.081
hsa-let-7a-5p	*TP53*	13-25	0.583	5'UTR	-15.400	-8.307	0.497	1.000	0.066
hsa-let-7a-5p	*TP53*	13-29	0.582	5'UTR	-19.200	-8.139	0.495	0.969	0.066
hsa-miR-25-3p	*TP53*	13-30	0.540	5'UTR	-17.300	-3.805	0.481	0.953	0.066
hsa-miR-31-5p	*TP53*	209-228	0.557	5'UTR	-18.300	-1.262	0.467	0.039	1.061
hsa-let-7a-5p	*TP53*	13-31	0.605	5'UTR	-26.700	-12.729	0.465	0.944	0.066
hsa-miR-15b-5p	*TP53*	07-30	0.537	5'UTR	-16.100	-0.484	0.458	0.957	0.046
hsa-miR-31-5p	*TP53*	12-28	0.612	5'UTR	-21	-8.837	0.458	0.995	0.051

hsa-miR-22-3p	*TP53*	199-218	0.534	5'UTR	-18.100	2.474	0.451	0.281	1.010
hsa-miR-93-5p	*TP53*	127-142	0.634	5'UTR	-17.300	-5.357	0.437	0.466	0.645
hsa-miR-200c-3p	*TP53*	125-139	0.519	5'UTR	-15.800	3.417	0.433	0.310	0.635
hsa-miR-31-5p	*TP53*	10-30	0.615	5'UTR	-26.100	-9.685	0.427	0.951	0.051
hsa-miR-200c-3p	*TP53*	128-146	0.579	5'UTR	-18.000	-1.580	0.409	0.581	0.650
hsa-miR-25-3p	*TP53*	84-100	0.577	5'UTR	-15.900	-3.798	0.406	0.228	0.426
hsa-miR-15b-5p	*TP53*	221-235	0.672	5'UTR	-17.300	-6.940	0.403	0.234	1.122

* Start and end position of the target region (site) predicted to be bound by miRNA; [a] Probability of the site being an miRNA binding site as predicted by our nonlinear logistic model; [b] A measure of stability for miRNA:target hybrid as computed by RNAhybrid; [c] A measure of the total energy change of the hybridization; [d] A measure of structural accessibility as computed by the average probability of a nucleotide being single-stranded (i.e., unpaired) for the nucleotides in the predicted binding site; [e] Conservation score by the PhastCons program for the binding site; [j] Relative starting location of the predicted binding site along the length of the sequence (e.g., for 3″ UTR, 0 indicates the 5″ end of the UTR, and 1 corresponds to the 3″ end).

Supplementary Table S2C. miRNA binding *'seedless'* regions identified in *RB1* gene.

miRNA	Gene	Site Position	LogitProb†	Region	ΔG_{hybrid}	ΔG_{total}*	Site_Access*	Site Consv*	Site Location*
hsa-miR-130a-3p	*RB1*	2529-2537	0.917	CDS	-17.100	-11.446	0.708	1.000	0.848
hsa-miR-31-5p	*RB1*	4704-4721	0.912	3'UTR	-20.100	-17.244	0.651	1.000	0.963
hsa-let-7a-5p	*RB1*	4686-4712	0.911	3'UTR	-22.100	-17.127	0.608	0.999	0.953
hsa-miR-31-5p	*RB1*	4704-4719	0.899	3'UTR	-18.700	-16.076	0.627	1000	0.963
hsa-miR-31-5p	*RB1*	4704-4717	0.898	3'UTR	-17.200	-14.545	0.672	1.000	0.963
hsa-let-7a-5p	*RB1*	4686-4719	0.896	3'UTR	-20.700	-14.420	0.573	1.000	0.953
hsa-miR-130a-3p	*RB1*	2529-2534	0.892	CDS	-15.900	-10.778	0.646	1.000	0.848
hsa-miR-142-3p	*RB1*	2002-2026	0.883	CDS	-18.500	-12.601	0.692	0.847	0.659
hsa-let-7a-5p	*RB1*	562-571	0.878	CDS	-15.500	-14.118	0.813	0.957	0.142
hsa-miR-200c-3p	*RB1*	3398-3414	0.878	3'UTR	-16.300	-14.615	0.846	0.809	0.245
hsa-miR-93-5p	*RB1*	2496-2518	0.863	CDS	-18.900	-14.159	0.741	0.998	0.836
hsa-miR-205-5p	*RB1*	1997-2016	0.861	CDS	-16.900	-10.178	0.715	0.997	0.657
hsa-miR-93-5p	*RB1*	4502-4513	0.860	3'UTR	-21.200	-17.937	0.510	0.891	0.852
hsa-miR-21-5p	*RB1*	579-608	0.859	CDS	-19.800	-13.221	0.582	0.999	0.148
hsa-miR-31-5p	*RB1*	4704-4739	0.857	3'UTR	-17.100	-11.413	0.501	0.997	0.963
hsa-miR-142-3p	*RB1*	1953-1972	0.854	CDS	-18.500	-10.406	0.665	0.987	0.641
hsa-miR-31-5p	*RB1*	4659-4676	0.854	3'UTR	-17.400	-11.832	0.614	0.993	0.938
hsa-miR-130a-3p	*RB1*	2767-2782	0.852	CDS	-16.400	-8.072	0.653	0.961	0.933
hsa-miR-223-3p	*RB1*	3583-3589	0.852	3'UTR	-11.700	-8.863	0.438	0.964	0.346
hsa-miR-223-3p	*RB1*	1784-1795	0.850	CDS	-17.400	-11.874	0.550	1000	0.581
hsa-miR-93-5p	*RB1*	4502-4517	0.850	3'UTR	-23.900	-20.873	0.555	0.771	0.852
hsa-miR-93-5p	*RB1*	725-738	0.849	CDS	-19.800	-16.717	0.628	0.733	0.201
hsa-miR-93-5p	*RB1*	4502-4511	0.848	3'UTR	-18.300	-14.488	0.471	0.939	0.852
hsa-miR-15b-5p	*RB1*	588-608	0.846	CDS	-17.500	-9.238	0.611	1000	0.151
hsa-miR-93-5p	*RB1*	4702-4719	0.846	3'UTR	-10.900	-8.169	0.658	1000	0.962
hsa-miR-93-5p	*RB1*	1232-1247	0.843	CDS	-15.600	-12.313	0.719	0.923	0.382
hsa-miR-130a-3p	*RB1*	4658-4680	0.842	3'UTR	-18.700	-10.840	0.599	0.991	0.937
hsa-miR-223-3p	*RB1*	4675-4694	0.842	3'UTR	-16.100	-8.561	0.470	0.999	0.947
hsa-let-7a-5p	*RB1*	2763-2773	0.841	CDS	-16.400	-9.961	0.485	0.999	0.932

hsa-miR-223-3p	*RB1*	773-792	0.840	CDS	-18.900	-12.323	0.472	0.999	0.218
hsa-miR-31-5p	*RB1*	4680-4706	0.838	3'UTR	-15.800	-8.671	0.538	1.000	0.949
hsa-let-7a-5p	*RB1*	4653-4671	0.836	3'UTR	-14.900	-9.896	0.683	0.882	0.935
hsa-let-7a-5p	*RB1*	2763-2778	0.835	CDS	-16.600	-9.837	0.601	0.999	0.932
hsa-let-7a-5p	*RB1*	4400-4414	0.832	3'UTR	-10.900	-9.056	0.412	0.899	0.795
hsa-miR-142-3p	*RB1*	4672-4694	0.831	3'UTR	-16.900	-9.585	0.501	0.999	0.945
hsa-miR-142-3p	*RB1*	585-609	0.829	CDS	-17.500	-10.442	0.545	1.000	0.150
hsa-miR-130a-3p	*RB1*	4658-4678	0.826	3'UTR	-14.600	-9.145	0.622	0.990	0.937
hsa-miR-200c-3p	*RB1*	4652-4678	0.826	3'UTR	-16.200	-10.719	0.629	0.906	0.934
hsa-miR-200c-3p	*RB1*	4652-4680	0.825	3'UTR	-18.600	-10.703	0.611	0.912	0.934
hsa-miR-130a-3p	*RB1*	2209-2224	0.824	CDS	-18.600	-10.320	0.577	1000	0.733
hsa-miR-21-5p	*RB1*	1348-1368	0.824	CDS	-18.800	-11.239	0.480	0.999	0.424
hsa-miR-142-3p	*RB1*	4074-4090	0.821	3'UTR	-17.500	-10.351	0.506	0.787	0.616
hsa-miR-142-3p	*RB1*	1953-1970	0.820	CDS	-12.300	-3.732	0.733	0.986	0.641
hsa-miR-200c-3p	*RB1*	1953-1973	0.820	CDS	-15.300	-6.921	0.635	0.987	0.641
hsa-miR-31-5p	*RB1*	4322-4356	0.818	3'UTR	-22.900	-7.727	0.444	0.950	0.753
hsa-let-7a-5p	*RB1*	4724-4745	0.817	3'UTR	-14.500	-6.891	0.464	0.996	0.974
hsa-miR-16-5p	*RB1*	4270-4295	0.817	3'UTR	-16.400	-8.430	0.460	0.998	0.724
hsa-miR-16-5p	*RB1*	3317-3339	0.817	3'UTR	-16.100	-7.382	0.404	0.905	0.200
hsa-miR-205-5p	*RB1*	1685-1692	0.817	CDS	-16.400	-8.626	0.703	0.997	0.545
hsa-miR-93-5p	*RB1*	1232-1253	0.817	CDS	-16.300	-10.122	0.643	0.829	0.382
hsa-miR-205-5p	*RB1*	3850-3864	0.816	3'UTR	-17.200	-6.314	0.436	0.912	0.493
hsa-miR-130a-3p	*RB1*	4329-4350	0.815	3'UTR	-15.300	-6.192	0.430	0.938	0.756
hsa-miR-205-5p	*RB1*	2136-2149	0.815	CDS	-15.100	-7.402	0.513	0.926	0.707
hsa-miR-25-3p	*RB1*	966-981	0.815	CDS	-15.900	-7.580	0.592	0.999	0.287
hsa-miR-93-5p	*RB1*	2496-2512	0.813	CDS	-16.300	-11.509	0.705	0.997	0.836
hsa-miR-223-3p	*RB1*	773-789	0.812	CDS	-17.600	-9.791	0.498	0.999	0.218
hsa-miR-93-5p	*RB1*	1223-1238	0.812	CDS	-15.200	-10.852	0.725	0.824	0.379
hsa-miR-93-5p	*RB1*	2097-2117	0.811	CDS	-10.800	-4.291	0.619	1000	0.693
hsa-miR-142-3p	*RB1*	4300-4315	0.808	3'UTR	-16.900	-7.407	0.591	0.997	0.741
hsa-miR-200c-3p	*RB1*	4078-4093	0.808	3'UTR	-17.900	-7.169	0.522	0.979	0.618

hsa-let-7a-5p	*RB1*	3410-3431	0.807	3'UTR	-20.400	-15.626	0.616	0.454	0.251
hsa-miR-25-3p	*RB1*	1783-1797	0.807	CDS	-17.300	-8.636	0.527	1000	0.580
hsa-miR-22-3p	*RB1*	4073-4093	0.804	3'UTR	-19.900	-9.273	0.459	0.770	0.616
hsa-miR-31-5p	*RB1*	725-738	0.804	CDS	-17.100	-14.017	0.628	0.733	0.201
hsa-miR-31-5p	*RB1*	2133-2151	0.804	CDS	-22.600	-14.890	0.533	0.899	0.706
hsa-miR-142-3p	*RB1*	4325-4350	0.803	3'UTR	-16.700	-8.095	0.497	0.934	0.754
hsa-miR-16-5p	*RB1*	585-608	0.803	CDS	-16.300	-9.267	0.560	1000	0.150
hsa-miR-93-5p	*RB1*	2913-2925	0.803	CDS	-15.900	-11.693	0.522	0.949	0.986
hsa-let-7a-5p	*RB1*	1122-1145	0.802	CDS	-16.900	-11.110	0.531	0.993	0.343
hsa-miR-200c-3p	*RB1*	2521-2532	0.802	CDS	-15.800	-9.390	0.569	1000	0.845
hsa-miR-142-3p	*RB1*	4074-4093	0.800	3'UTR	-17.900	-7.394	0.481	0.807	0.616
hsa-miR-200c-3p	*RB1*	3613-3625	0.800	3'UTR	-15.300	-8.792	0.518	0.970	0.363
hsa-miR-15b-5p	*RB1*	1245-1256	0.799	CDS	-16.200	-11.129	0.558	0.764	0.387
hsa-miR-93-5p	*RB1*	3370-3382	0.799	3'UTR	-15.800	-11.245	0.573	0.283	0.229
hsa-miR-93-5p	*RB1*	3858-3875	0.799	3'UTR	-16.100	-12.674	0.546	0.761	0.498
hsa-miR-200c-3p	*RB1*	1953-1976	0.798	CDS	-16.600	-3.940	0.622	0.989	0.641
hsa-let-7a-5p	*RB1*	3410-3429	0.797	3'UTR	-18.400	-13.675	0.623	0.500	0.251
hsa-let-7a-5p	*RB1*	4724-4743	0.796	3'UTR	-11.800	-4.428	0.460	0.996	0.974
hsa-miR-142-3p	*RB1*	3603-3624	0.795	3'UTR	-16.600	-10.934	0.514	0.690	0.357
hsa-miR-22-3p	*RB1*	474-486	0.794	CDS	-19.100	-11.241	0.615	0.999	0.111
hsa-miR-93-5p	*RB1*	4666-4693	0.793	3'UTR	-15.500	-5.368	0.510	0.995	0.942
hsa-miR-15b-5p	*RB1*	720-739	0.792	CDS	-16.300	-12.693	0.654	0.813	0.199
hsa-miR-15b-5p	*RB1*	3409-3428	0.791	3'UTR	-16.900	-12.241	0.618	0.550	0.251
hsa-miR-31-5p	*RB1*	3128-3142	0.790	3'UTR	-17.400	-12.125	0.542	0.004	0.096
hsa-miR-130a-3p	*RB1*	722-733	0.789	CDS	-14.300	-11.270	0.763	0.689	0.199
hsa-miR-200c-3p	*RB1*	585-597	0.789	CDS	-15.400	-9.279	0.557	1.000	0.150
hsa-miR-16-5p	*RB1*	4736-4762	0.788	3'UTR	-12.900	-4.291	0.477	0.998	0.980
hsa-miR-16-5p	*RB1*	4310-4325	0.788	3'UTR	-15.600	-6.496	0.426	0.995	0.746
hsa-miR-130a-3p	*RB1*	3118-3140	0.787	3'UTR	-18.400	-12.682	0.519	0.041	0.091
hsa-let-7a-5p	*RB1*	4542-4554	0.786	3'UTR	-16.200	-10.299	0.530	0.977	0.874
hsa-miR-16-5p	*RB1*	4310-4332	0.786	3'UTR	-17.800	-6.907	0.432	0.931	0.746
hsa-let-7a-5p	*RB1*	3899-3921	0.785	3'UTR	-16.600	-5.382	0.452	0.961	0.520

hsa-miR-15b-5p	*RB1*	326-342	0.785	CDS	-18.900	-7.844	0.520	0.999	0.057
hsa-let-7a-5p	*RB1*	582-613	0.784	CDS	-17.500	-10.650	0.522	0.999	0.149
hsa-miR-200c-3p	*RB1*	4360-4395	0.784	3'UTR	-17.800	-6.654	0.403	0.938	0.774
hsa-miR-200c-3p	*RB1*	891-901	0.784	CDS	-16.200	-5.948	0.501	0.997	0.260
hsa-let-7a-5p	*RB1*	2528-2551	0.783	CDS	-16.100	-7.441	0.560	0.999	0.848
hsa-miR-31-5p	*RB1*	3584-3604	0.783	3'UTR	-17.600	-4.768	0.404	0.675	0.347
hsa-let-7a-5p	*RB1*	2528-2557	0.782	CDS	-16.800	-8.133	0.598	0.999	0.848
hsa-let-7a-5p	*RB1*	1883-1901	0.782	CDS	-15.100	-8.688	0.479	0.995	0.616
hsa-miR-16-5p	*RB1*	704-729	0.782	CDS	-15.300	-8.586	0.564	0.904	0.193
hsa-let-7a-5p	*RB1*	4644-4664	0.780	3'UTR	-17.500	-8.263	0.630	0.774	0.930
hsa-miR-22-3p	*RB1*	2014-2031	0.779	CDS	-15.400	-5.093	0.471	0.788	0.663
hsa-miR-16-5p	*RB1*	3411-3428	0.778	3'UTR	-15.900	-10.661	0.596	0.500	0.252
hsa-miR-200c-3p	*RB1*	4360-4393	0.778	3'UTR	-15.100	-5.847	0.421	0.934	0.774
hsa-let-7a-5p	*RB1*	3584-3604	0.776	3'UTR	-16.900	-4.068	0.404	0.675	0.347
hsa-miR-200c-3p	*RB1*	4490-4507	0.776	3'UTR	-18.400	-13.994	0.590	0.420	0.845
hsa-miR-25-3p	*RB1*	2371-2391	0.776	CDS	-17.800	-9.103	0.463	0.998	0.791
hsa-miR-200c-3p	*RB1*	2521-2540	0.775	CDS	-16.400	-8.935	0.603	1000	0.845
hsa-miR-22-3p	*RB1*	4304-4326	0.774	3'UTR	-19.700	-5.308	0.434	0.995	0.743
hsa-miR-25-3p	*RB1*	2371-2395	0.774	CDS	-19.600	-8.294	0.412	0.999	0.791
hsa-miR-130a-3p	*RB1*	2491-2507	0.773	CDS	-17.800	-10.470	0.664	0.998	0.834
hsa-miR-93-5p	*RB1*	1847-1872	0.773	CDS	-19.500	-4.158	0.408	0.998	0.603
hsa-miR-130a-3p	*RB1*	4077-4094	0.772	3'UTR	-16.200	-5.492	0.570	0.891	0.618
hsa-miR-223-3p	*RB1*	1784-1807	0.772	CDS	-18.300	-7.358	0.445	0.999	0.581
hsa-miR-93-5p	*RB1*	3456-3473	0.772	3'UTR	-17.400	-11.863	0.572	0.119	0.277
hsa-let-7a-5p	*RB1*	1294-1314	0.771	CDS	-16.800	-4.784	0.414	0.985	0.405
hsa-miR-142-3p	*RB1*	1205-1226	0.771	CDS	-20.700	-10.878	0.482	0.850	0.373
hsa-miR-21-5p	*RB1*	1348-1366	0.771	CDS	-15.100	-5.350	0.471	0.998	0.424
hsa-miR-25-3p	*RB1*	491-502	0.771	CDS	-18.600	-13.270	0.462	0.999	0.117
hsa-miR-31-5p	*RB1*	4644-4662	0.771	3'UTR	-17.300	-7.854	0.598	0.750	0.930
hsa-miR-93-5p	*RB1*	1847-1868	0.771	CDS	-19.100	-4.499	0.450	0.998	0.603
hsa-miR-16-5p	*RB1*	1617-1633	0.770	CDS	-21.500	-11.348	0.460	0.939	0.521

hsa-miR-22-3p	*RB1*	4073-4097	0.770	3'UTR	-21.600	-6.282	0.431	0.720	0.616
hsa-let-7a-5p	*RB1*	4075-4093	0.769	3'UTR	-15.400	-4.868	0.503	0.848	0.617
hsa-miR-130a-3p	*RB1*	3968-3980	0.769	3'UTR	-16.500	-13.304	0.714	0.356	0.558
hsa-miR-223-3p	*RB1*	4307-4316	0.768	3'UTR	-15.400	-3.560	0.478	0.995	0.744
hsa-let-7a-5p	*RB1*	4542-4556	0.767	3'UTR	-20.100	-10.247	0.465	0.949	0.874
hsa-miR-130a-3p	*RB1*	3968-3973	0.767	3'UTR	-15.300	-11.859	0.644	0.473	0.558
hsa-miR-15b-5p	*RB1*	3115-3143	0.766	3'UTR	-18.100	-10.621	0.529	0.100	0.089
hsa-miR-200c-3p	*RB1*	2140-2150	0.766	CDS	-19.500	-9.771	0.641	0.908	0.708
hsa-miR-15b-5p	*RB1*	3115-3133	0.765	3'UTR	-16.900	-12.705	0.587	0.151	0.089
hsa-miR-25-3p	*RB1*	2371-2385	0.765	CDS	-16.300	-8.312	0.474	0.998	0.791
hsa-let-7a-5p	*RB1*	1122-1143	0.764	CDS	-12.400	-6.512	0.513	0.993	0.343
hsa-let-7a-5p	*RB1*	1049-1061	0.763	CDS	-12.900	-8.545	0.588	1000	0.317
hsa-miR-205-5p	*RB1*	2558-2565	0.763	CDS	-15.600	-8.224	0.444	0.999	0.858
hsa-miR-223-3p	*RB1*	576-584	0.761	CDS	-12.300	-7.623	0.576	0.999	0.147
hsa-miR-22-3p	*RB1*	508-525	0.761	CDS	-16.900	-8.978	0.449	0.997	0.123
hsa-miR-31-5p	*RB1*	339-350	0.761	CDS	-16.300	-7.200	0.405	1	0.062
hsa-miR-93-5p	*RB1*	1056-1069	0.761	CDS	-15.100	-8.541	0.420	1000	0.319
hsa-miR-223-3p	*RB1*	4443-4461	0.760	3'UTR	-17.100	-6.904	0.402	0.777	0.819
hsa-miR-22-3p	*RB1*	1955-1974	0.760	CDS	-18.800	-9.964	0.616	0.997	0.642
hsa-miR-25-3p	*RB1*	491-506	0.760	CDS	-20.300	-13.842	0.432	0.999	0.117
hsa-miR-15b-5p	*RB1*	3115-3131	0.759	3'UTR	-15.300	-11.698	0.586	0.168	0.089
hsa-miR-16-5p	*RB1*	704-739	0.759	CDS	-16.300	-8.274	0.569	0.892	0.193
hsa-miR-21-5p	*RB1*	3127-3143	0.759	3'UTR	-15.800	-9.819	0.544	0.004	0.096
hsa-miR-31-5p	*RB1*	1778-1794	0.759	CDS	-17.700	-6.670	0.515	1000	0.578
hsa-miR-93-5p	*RB1*	2386-2413	0.759	CDS	-15.400	-3.874	0.433	0.956	0.797
hsa-miR-205-5p	*RB1*	1280-1295	0.758	CDS	-17.100	-9.382	0.433	1000	0.400
hsa-miR-93-5p	*RB1*	4261-4280	0.758	3'UTR	-15.600	-7.220	0.430	1	0.719
hsa-let-7a-5p	*RB1*	3410-3426	0.757	3'UTR	-15.600	-10.896	0.582	0.588	0.251
hsa-miR-223-3p	*RB1*	4075-4093	0.757	3'UTR	-15.200	-4.668	0.503	0.848	0.617
hsa-let-7a-5p	*RB1*	2344-2364	0.755	CDS	-18.700	-4.799	0.409	0.956	0.781
hsa-miR-130a-3p	*RB1*	2209-2232	0.755	CDS	-19.100	-1.589	0.515	0.984	0.733
hsa-miR-223-3p	*RB1*	3415-3429	0.755	3'UTR	-16.600	-12.111	0.599	0.333	0.254

hsa-miR-31-5p	*RB1*	3383-3407	0.755	3'UTR	-17.200	-5.644	0.541	0.621	0.236
hsa-miR-93-5p	*RB1*	1771-1791	0.755	CDS	-16.800	-7.883	0.425	1000	0.576
hsa-miR-31-5p	*RB1*	2086-2102	0.754	CDS	-15.400	-9.036	0.637	0.796	0.689
hsa-miR-93-5p	*RB1*	2883-2898	0.754	CDS	-16.800	-4.456	0.453	0.994	0.975
hsa-miR-16-5p	*RB1*	3118-3143	0.753	3'UTR	-15.200	-8.595	0.546	0.036	0.091
hsa-miR-21-5p	*RB1*	692-709	0.753	CDS	-17.500	-2.897	0.445	0.993	0.189
hsa-miR-21-5p	*RB1*	704-739	0.752	CDS	-18.500	-10.474	0.569	0.892	0.193
hsa-miR-15b-5p	*RB1*	488-526	0.751	CDS	-17.400	-6.698	0.434	0.980	0.116
hsa-miR-205-5p	*RB1*	4297-4320	0.749	3'UTR	-15.700	-3.248	0.591	0.998	0.739
hsa-miR-25-3p	*RB1*	2445-2456	0.749	CDS	-15.400	-5.139	0.509	1	0.818
hsa-miR-31-5p	*RB1*	605-613	0.749	CDS	-18.200	-10.253	0.472	0.997	0.158
hsa-miR-16-5p	*RB1*	4643-4669	0.748	3'UTR	-15.700	-4.893	0.604	0.783	0.929
hsa-miR-93-5p	*RB1*	583-607	0.748	CDS	-16.700	-9.956	0.575	1000	0.150
hsa-miR-142-3p	*RB1*	769-784	0.747	CDS	-16.100	-8.685	0.429	0.999	0.216
hsa-miR-200c-3p	*RB1*	4078-4097	0.747	3'UTR	-18.100	-2.677	0.473	0.874	0.618
hsa-miR-21-5p	*RB1*	1790-1808	0.747	CDS	-15.500	-6.582	0.432	0.999	0.583
hsa-miR-21-5p	*RB1*	692-713	0.747	CDS	-18.100	-2.166	0.430	0.993	0.189
hsa-miR-21-5p	*RB1*	482-502	0.747	CDS	-17.400	-5.651	0.411	0.999	0.113
hsa-let-7a-5p	*RB1*	3960-3978	0.746	3'UTR	-16.200	-13.257	0.666	0.342	0.554
hsa-miR-130a-3p	*RB1*	4415-4432	0.746	3'UTR	-16.400	-6.311	0.439	0.458	0.804
hsa-miR-200c-3p	*RB1*	4490-4505	0.746	3'UTR	-16.200	-12.685	0.593	0.347	0.845
hsa-miR-205-5p	*RB1*	1685-1715	0.746	CDS	-16.600	-5.737	0.455	0.999	0.545
hsa-miR-223-3p	*RB1*	3854-3874	0.746	3'UTR	-15.600	-5.290	0.451	0.842	0.495
hsa-miR-93-5p	*RB1*	1248-1261	0.746	CDS	-16.700	-9.155	0.488	0.746	0.388
hsa-let-7a-5p	*RB1*	325-343	0.745	CDS	-15.500	-3.901	0.512	1000	0.057
hsa-miR-130a-3p	*RB1*	546-570	0.745	CDS	-15.700	-2.087	0.441	0.982	0.136
hsa-miR-130a-3p	*RB1*	3176-3194	0.744	3'UTR	-19.100	-5.499	0.501	0.611	0.123
hsa-miR-31-5p	*RB1*	1778-1792	0.744	CDS	-15.900	-6.260	0.503	0.999	0.578
hsa-miR-31-5p	*RB1*	1689-1705	0.744	CDS	-15.200	-10.309	0.452	1000	0.546
hsa-miR-130a-3p	*RB1*	3390-3414	0.743	3'UTR	-15.100	-1.810	0.632	0.705	0.240
hsa-miR-15b-5p	*RB1*	2265-2291	0.743	CDS	-23.600	-10.426	0.476	1000	0.753

hsa-miR-15b-5p	*RB1*	2265-2285	0.743	CDS	-22.500	-8.239	0.450	1000	0.753
hsa-miR-31-5p	*RB1*	1931-1951	0.742	CDS	-17.600	-3.629	0.466	0.983	0.633
hsa-let-7a-5p	*RB1*	3597-3617	0.741	3'UTR	-19.800	-12.237	0.538	0.495	0.354
hsa-miR-142-3p	*RB1*	4594-4613	0.741	3'UTR	-15.500	-5.291	0.404	0.937	0.902
hsa-miR-15b-5p	*RB1*	2265-2283	0.741	CDS	-19.300	-7.928	0.473	1	0.753
hsa-let-7a-5p	*RB1*	2020-2043	0.740	CDS	-19.300	-7.459	0.509	0.825	0.665
hsa-miR-223-3p	*RB1*	3415-3427	0.740	3'UTR	-15.900	-11.398	0.553	0.384	0.254
hsa-miR-93-5p	*RB1*	583-610	0.740	CDS	-16.700	-9.911	0.557	1000	0.150
hsa-let-7a-5p	*RB1*	1906-1924	0.739	CDS	-17.600	-7.454	0.521	0.996	0.624
hsa-miR-205-5p	*RB1*	2376-2409	0.738	CDS	-16.800	-4.715	0.491	0.984	0.793
hsa-miR-223-3p	*RB1*	4249-4262	0.738	3'UTR	-15.200	-4.431	0.444	1000	0.712
hsa-miR-200c-3p	*RB1*	2542-2560	0.737	CDS	-16.500	-7.127	0.596	0.998	0.853
hsa-miR-25-3p	*RB1*	1195-1211	0.737	CDS	-18.400	-9.316	0.414	0.984	0.369
hsa-miR-16-5p	*RB1*	504-526	0.735	CDS	-15.800	-6.110	0.421	0.966	0.121
hsa-miR-200c-3p	*RB1*	3760-3773	0.735	3'UTR	-18.300	-10.193	0.498	0.663	0.444
hsa-miR-142-3p	*RB1*	3603-3616	0.734	3'UTR	-15.900	-10.034	0.519	0.519	0.357
hsa-miR-31-5p	*RB1*	4071-4083	0.734	3'UTR	-19.600	-9.022	0.482	0.494	0.615
hsa-miR-93-5p	*RB1*	4072-4094	0.734	3'UTR	-15.100	-4.439	0.463	0.704	0.615
hsa-miR-93-5p	*RB1*	1771-1787	0.734	CDS	-15.400	-5.827	0.417	1000	0.576
hsa-let-7a-5p	*RB1*	3495-3523	0.731	3'UTR	-11.400	-1.521	0.419	0.791	0.298
hsa-miR-22-3p	*RB1*	699-738	0.731	CDS	-20.900	-8.015	0.509	0.903	0.191
hsa-miR-130a-3p	*RB1*	4642-4667	0.730	3'UTR	-15.500	-3.954	0.592	0.755	0.929
hsa-miR-130a-3p	*RB1*	4071-4090	0.730	3'UTR	-16.100	-5.234	0.434	0.671	0.615
hsa-miR-93-5p	*RB1*	1157-1175	0.730	CDS	-13.200	-2.917	0.403	0.947	0.356
hsa-miR-200c-3p	*RB1*	1953-1980	0.729	CDS	-16.800	0.195	0.563	0.991	0.641
hsa-miR-21-5p	*RB1*	2131-2152	0.729	CDS	-15.100	-6.424	0.514	0.907	0.705
hsa-miR-31-5p	*RB1*	3128-3139	0.729	3'UTR	-15.400	-10.354	0.436	0.005	0.096
hsa-miR-93-5p	*RB1*	1965-1976	0.728	CDS	-17.200	-4.059	0.419	0.999	0.645
hsa-miR-22-3p	*RB1*	2110-2136	0.727	CDS	-15.100	-4.077	0.435	0.963	0.698
hsa-miR-25-3p	*RB1*	1527-1541	0.727	CDS	-16.100	-2.878	0.410	1	0.488
hsa-miR-93-5p	*RB1*	2621-2635	0.726	CDS	-16.700	-5.039	0.466	0.907	0.881
hsa-miR-15b-5p	*RB1*	1245-1264	0.725	CDS	-16.300	-8.302	0.486	0.659	0.387

hsa-miR-93-5p	*RB1*	3174-3193	0.725	3'UTR	-16.700	-4.975	0.520	0.595	0.121
hsa-let-7a-5p	*RB1*	3597-3614	0.723	3'UTR	-17.300	-9.745	0.598	0.418	0.354
hsa-miR-130a-3p	*RB1*	1616-1636	0.723	CDS	-23.900	-13.349	0.413	0.951	0.520
hsa-miR-15b-5p	*RB1*	488-506	0.723	CDS	-15.900	-8.912	0.408	0.999	0.116
hsa-miR-31-5p	*RB1*	481-495	0.723	CDS	-18.600	-10.070	0.450	1.000	0.113
hsa-miR-31-5p	*RB1*	2125-2147	0.722	CDS	-20.400	-1.937	0.489	0.913	0.703
hsa-miR-93-5p	*RB1*	3757-3772	0.722	3'UTR	-17.600	-9.883	0.448	0.529	0.442
hsa-let-7a-5p	*RB1*	3389-3405	0.721	3'UTR	-17.300	-4.982	0.523	0.543	0.240
hsa-let-7a-5p	*RB1*	1912-1940	0.721	CDS	-23.500	-5.665	0.472	0.977	0.626
hsa-miR-223-3p	*RB1*	4450-4470	0.721	3'UTR	-17.400	-7.869	0.504	0.538	0.823
hsa-miR-25-3p	*RB1*	2445-2465	0.719	CDS	-16.100	-7.374	0.573	1	0.818
hsa-miR-31-5p	*RB1*	3062-3075	0.718	3'UTR	-16.700	-13.797	0.597	0.077	0.060
hsa-let-7a-5p	*RB1*	691-715	0.717	CDS	-15.100	1.725	0.461	0.994	0.188
hsa-miR-130a-3p	*RB1*	3390-3419	0.717	3'UTR	-15.100	1.004	0.600	0.753	0.240
hsa-miR-31-5p	*RB1*	2473-2505	0.717	CDS	-19.800	1.294	0.450	0.975	0.828
hsa-miR-93-5p	*RB1*	1682-1697	0.716	CDS	-17.700	-8.893	0.479	0.994	0.544
hsa-miR-15b-5p	*RB1*	2265-2281	0.715	CDS	-15.300	-6.150	0.497	1	0.753
hsa-miR-200c-3p	*RB1*	914-930	0.715	CDS	-19.900	-7.796	0.545	1.000	0.268
hsa-miR-223-3p	*RB1*	3411-3425	0.715	3'UTR	-12.500	-7.230	0.557	0.600	0.252
hsa-miR-93-5p	*RB1*	328-348	0.714	CDS	-20.300	-6.365	0.441	1000	0.058
hsa-let-7a-5p	*RB1*	887-902	0.713	CDS	-17.800	-5.822	0.468	0.997	0.259
hsa-miR-142-3p	*RB1*	323-343	0.713	CDS	-14.200	-1.006	0.512	0.999	0.056
hsa-miR-130a-3p	*RB1*	4485-4505	0.712	3'UTR	-15.300	-12.104	0.620	0.278	0.842
hsa-miR-25-3p	*RB1*	2018-2033	0.712	CDS	-15.200	-9.630	0.622	0.766	0.665
hsa-miR-93-5p	*RB1*	1682-1702	0.712	CDS	-20.100	-11.296	0.565	0.995	0.544
hsa-let-7a-5p	*RB1*	2126-2146	0.711	CDS	-19.100	-1.328	0.485	0.905	0.703
hsa-miR-21-5p	*RB1*	3127-3136	0.711	3'UTR	-15.300	-7.819	0.540	0.006	0.096
hsa-miR-93-5p	*RB1*	412-427	0.711	CDS	-15.100	-7.548	0.404	0.991	0.088
hsa-miR-142-3p	*RB1*	549-571	0.709	CDS	-15.700	-1.508	0.507	0.980	0.137
hsa-miR-142-3p	*RB1*	2073-2100	0.709	CDS	-15.100	-4.927	0.454	0.831	0.684
hsa-miR-205-5p	*RB1*	767-792	0.709	CDS	-16.400	-5.492	0.435	0.999	0.216

hsa-let-7a-5p	*RB1*	481-495	0.708	CDS	-17.200	-8.670	0.450	1000	0.113
hsa-miR-15b-5p	*RB1*	1245-1269	0.708	CDS	-17.400	-10.267	0.538	0.548	0.387
hsa-miR-200c-3p	*RB1*	2285-2297	0.708	CDS	-15.900	-1.198	0.436	1000	0.760
hsa-miR-93-5p	*RB1*	1056-1072	0.707	CDS	-15.300	-2.558	0.405	1000	0.319
hsa-miR-15b-5p	*RB1*	625-636	0.705	CDS	-16.600	-10.520	0.484	0.995	0.165
hsa-miR-15b-5p	*RB1*	625-640	0.704	CDS	-16.800	-10.715	0.588	0.990	0.165
hsa-miR-93-5p	*RB1*	2204-2224	0.704	CDS	-21.400	-10.918	0.509	1.000	0.731
hsa-miR-93-5p	*RB1*	1518-1534	0.703	CDS	-16.400	-9.759	0.527	0.998	0.485
hsa-let-7a-5p	*RB1*	3015-3032	0.700	3'UTR	-21.400	-13.943	0.509	0	0.034
hsa-miR-22-3p	*RB1*	2283-2297	0.700	CDS	-15.400	0.354	0.423	0.999	0.760
hsa-miR-93-5p	*RB1*	1652-1664	0.700	CDS	-16.400	-11.807	0.452	0.959	0.533
hsa-miR-130a-3p	*RB1*	4642-4660	0.699	3'UTR	-15.400	-4.185	0.511	0.666	0.929
hsa-miR-200c-3p	*RB1*	3760-3781	0.699	3'UTR	-21.400	-12.192	0.487	0.575	0.444
hsa-miR-200c-3p	*RB1*	2849-2873	0.699	CDS	-22.900	-5.899	0.400	0.996	0.963
hsa-miR-93-5p	*RB1*	328-343	0.699	CDS	-16.100	-2.657	0.498	0.999	0.058
hsa-miR-93-5p	*RB1*	1989-2023	0.698	CDS	-15.700	-7.639	0.648	0.930	0.654
hsa-let-7a-5p	*RB1*	3834-3852	0.697	3'UTR	-12.600	-0.684	0.495	0.900	0.484
hsa-let-7a-5p	*RB1*	3375-3400	0.697	3'UTR	-13.200	-1.045	0.451	0.478	0.232
hsa-miR-93-5p	*RB1*	628-648	0.697	CDS	-16.600	-13.582	0.537	0.992	0.166
hsa-miR-93-5p	*RB1*	328-345	0.697	CDS	-18.900	-3.359	0.455	1000	0.058
hsa-miR-93-5p	*RB1*	1619-1640	0.697	CDS	-16.700	-5.464	0.429	0.953	0.521
hsa-miR-93-5p	*RB1*	2319-2348	0.697	CDS	-19.100	-4.287	0.403	0.963	0.773
hsa-let-7a-5p	*RB1*	2020-2050	0.695	CDS	-19.600	-7.748	0.523	0.815	0.665
hsa-miR-16-5p	*RB1*	4071-4097	0.695	3'UTR	-16.500	-1.095	0.400	0.667	0.615
hsa-miR-21-5p	*RB1*	4401-4429	0.695	3'UTR	-10.900	-3.008	0.400	0.639	0.796
hsa-miR-223-3p	*RB1*	683-701	0.694	CDS	-16.600	-2.385	0.499	0.999	0.186
hsa-miR-93-5p	*RB1*	711-732	0.693	CDS	-15.500	-8.784	0.589	0.830	0.196
hsa-let-7a-5p	*RB1*	3756-3772	0.692	3'UTR	-16.200	-8.167	0.426	0.503	0.441
hsa-miR-21-5p	*RB1*	4071-4084	0.692	3'UTR	-15.500	-4.978	0.517	0.530	0.615
hsa-miR-25-3p	*RB1*	2208-2233	0.692	CDS	-16.900	-3.659	0.484	0.986	0.733
hsa-miR-21-5p	*RB1*	2274-2296	0.691	CDS	-16.500	-1.124	0.461	1.000	0.756
hsa-miR-21-5p	*RB1*	502-526	0.691	CDS	-16.200	-4.253	0.427	0.969	0.121

hsa-miR-25-3p	*RB1*	586-610	0.690	CDS	-18.100	-6.217	0.538	1.000	0.151
hsa-miR-31-5p	*RB1*	2022-2043	0.690	CDS	-18.200	-6.301	0.471	0.819	0.666
hsa-miR-142-3p	*RB1*	1251-1282	0.688	CDS	-15.300	-6.634	0.594	0.694	0.389
hsa-miR-31-5p	*RB1*	2440-2474	0.687	CDS	-15.400	3.127	0.400	0.975	0.816
hsa-miR-93-5p	*RB1*	4437-4458	0.687	3'UTR	-16.700	-6.903	0.414	0.592	0.816
hsa-miR-31-5p	*RB1*	4250-4269	0.684	3'UTR	-15.900	-2.345	0.413	1.000	0.713
hsa-miR-142-3p	*RB1*	710-733	0.681	CDS	-10.700	-6.789	0.574	0.843	0.195
hsa-miR-25-3p	*RB1*	586-606	0.680	CDS	-16.600	-4.770	0.536	1000	0.151
hsa-miR-142-3p	*RB1*	1251-1288	0.679	CDS	-17.100	-7.087	0.594	0.742	0.389
hsa-miR-93-5p	*RB1*	683-701	0.679	CDS	-18.100	-3.885	0.499	0.999	0.186
hsa-miR-31-5p	*RB1*	605-636	0.678	CDS	-21.600	-4.605	0.419	0.982	0.158
hsa-let-7a-5p	*RB1*	3015-3030	0.677	3'UTR	-19.200	-13.083	0.545	0	0.034
hsa-miR-22-3p	*RB1*	2860-2871	0.677	CDS	-17.700	-2.417	0.414	1000	0.967
hsa-miR-200c-3p	*RB1*	4490-4502	0.675	3'UTR	-15.300	-12.258	0.592	0.200	0.845
hsa-miR-200c-3p	*RB1*	2849-2863	0.675	CDS	-15.600	-6.395	0.432	0.994	0.963
hsa-let-7a-5p	*RB1*	1912-1934	0.674	CDS	-18.200	-4.841	0.553	0.976	0.626
hsa-miR-31-5p	*RB1*	2125-2145	0.674	CDS	-18.300	0.163	0.441	0.905	0.703
hsa-miR-93-5p	*RB1*	4643-4663	0.674	3'UTR	-12.800	-2.504	0.590	0.727	0.929
hsa-miR-130a-3p	*RB1*	4450-4472	0.673	3'UTR	-16.200	-5.614	0.469	0.525	0.823
hsa-miR-142-3p	*RB1*	3748-3774	0.672	3'UTR	-16.400	-5.891	0.408	0.428	0.437
hsa-miR-21-5p	*RB1*	2573-2599	0.672	CDS	-16.100	-2.854	0.487	0.990	0.864
hsa-miR-200c-3p	*RB1*	690-701	0.671	CDS	-16.400	-1.064	0.431	0.999	0.188
hsa-miR-22-3p	*RB1*	1381-1392	0.671	CDS	-16.500	-1.720	0.460	1000	0.436
hsa-miR-25-3p	*RB1*	1248-1268	0.670	CDS	-16.100	-8.522	0.510	0.498	0.388
hsa-miR-93-5p	*RB1*	412-423	0.669	CDS	-15.100	-7.620	0.516	1000	0.088
hsa-miR-93-5p	*RB1*	1899-1925	0.669	CDS	-15.100	-3.195	0.504	0.994	0.622
hsa-let-7a-5p	*RB1*	4470-4493	0.668	3'UTR	-17.200	-8.168	0.412	0.347	0.834
hsa-miR-130a-3p	*RB1*	3566-3588	0.668	3'UTR	-16.800	-4.144	0.478	0.401	0.337
hsa-miR-22-3p	*RB1*	2125-2151	0.668	CDS	-18.100	0.363	0.419	0.925	0.703
hsa-miR-25-3p	*RB1*	2940-2954	0.667	CDS	-15.200	-3.888	0.421	0.985	0.995
hsa-miR-130a-3p	*RB1*	1616-1633	0.665	CDS	-19.200	-8.950	0.456	0.943	0.520

hsa-miR-142-3p	*RB1*	4092-4111	0.665	3'UTR	-11.200	-0.869	0.595	0.631	0.626
hsa-miR-93-5p	*RB1*	2769-2798	0.665	CDS	-16.900	-3.497	0.619	0.958	0.934
hsa-miR-93-5p	*RB1*	683-704	0.665	CDS	-19.800	-0.447	0.436	0.998	0.186
hsa-miR-200c-3p	*RB1*	474-489	0.662	CDS	-18.300	-6.268	0.565	0.999	0.111
hsa-miR-22-3p	*RB1*	3059-3077	0.662	3'UTR	-17.400	-8.706	0.491	0.066	0.058
hsa-let-7a-5p	*RB1*	849-872	0.661	CDS	-15.500	1.903	0.441	0.990	0.245
hsa-miR-130a-3p	*RB1*	3118-3138	0.661	3'UTR	-15.400	-5.500	0.509	0.045	0.091
hsa-let-7a-5p	*RB1*	1912-1936	0.659	CDS	-19.400	-5.974	0.511	0.977	0.626
hsa-miR-15b-5p	*RB1*	3167-3193	0.659	3'UTR	-16.400	2.472	0.401	0.611	0.118
hsa-miR-205-5p	*RB1*	3063-3078	0.659	3'UTR	-17.900	-9.230	0.491	0.086	0.060
hsa-miR-25-3p	*RB1*	1253-1272	0.659	CDS	-15.100	-6.687	0.597	0.558	0.390
hsa-miR-25-3p	*RB1*	700-733	0.659	CDS	-15.700	-0.958	0.520	0.886	0.192
hsa-let-7a-5p	*RB1*	4087-4114	0.658	3'UTR	-14.700	-0.586	0.535	0.633	0.623
hsa-miR-142-3p	*RB1*	3112-3133	0.658	3'UTR	-10.900	-4.289	0.581	0.223	0.087
hsa-miR-142-3p	*RB1*	900-930	0.658	CDS	-16.300	-0.721	0.578	1000	0.263
hsa-miR-93-5p	*RB1*	412-429	0.658	CDS	-17.900	-5.551	0.415	0.987	0.088
hsa-miR-130a-3p	*RB1*	3390-3427	0.656	3'UTR	-15.500	1.660	0.605	0.595	0.240
hsa-miR-21-5p	*RB1*	1911-1933	0.656	CDS	-15.300	-1.841	0.556	0.976	0.626
hsa-miR-142-3p	*RB1*	460-468	0.655	CDS	-15.200	-2.439	0.447	0.988	0.105
hsa-miR-22-3p	*RB1*	3059-3067	0.655	3'UTR	-16.600	-7.933	0.408	0.028	0.058
hsa-miR-93-5p	*RB1*	467-487	0.655	CDS	-21.600	-11.060	0.524	0.999	0.108
hsa-let-7a-5p	*RB1*	2020-2041	0.654	CDS	-16.400	-1.868	0.554	0.830	0.665
hsa-miR-16-5p	*RB1*	2025-2049	0.654	CDS	-16.600	-4.600	0.458	0.850	0.667
hsa-miR-31-5p	*RB1*	1449-1459	0.654	CDS	-11.700	-7.117	0.499	0.998	0.460
hsa-miR-25-3p	*RB1*	2906-2924	0.653	CDS	-15.300	-1.973	0.469	0.960	0.983
hsa-miR-130a-3p	*RB1*	3065-3079	0.651	3'UTR	-16.500	-6.666	0.520	0.093	0.062
hsa-miR-31-5p	*RB1*	3025-3048	0.651	3'UTR	-21.200	-7.159	0.417	0.015	0.040
hsa-miR-93-5p	*RB1*	467-490	0.651	CDS	-22.900	-10.497	0.494	0.999	0.108
hsa-miR-25-3p	*RB1*	1963-1983	0.649	CDS	-15.700	0.280	0.430	0.998	0.645
hsa-miR-31-5p	*RB1*	2218-2229	0.648	CDS	-15.200	-2.728	0.483	0.969	0.736
hsa-miR-142-3p	*RB1*	1251-1285	0.647	CDS	-15.600	-4.576	0.583	0.720	0.389
hsa-miR-25-3p	*RB1*	2264-2290	0.647	CDS	-16.600	-3.493	0.477	1000	0.753

hsa-miR-205-5p	*RB1*	3683-3703	0.646	3'UTR	-15.400	-6.537	0.479	0.139	0.401
hsa-miR-142-3p	*RB1*	2073-2114	0.645	CDS	-10.800	1.261	0.494	0.887	0.684
hsa-miR-15b-5p	*RB1*	1617-1636	0.645	CDS	-16.200	-5.748	0.414	0.948	0.521
hsa-miR-205-5p	*RB1*	2906-2928	0.645	CDS	-15.500	-0.960	0.476	0.960	0.983
hsa-let-7a-5p	*RB1*	1906-1920	0.643	CDS	-15.500	-5.329	0.426	0.997	0.624
hsa-miR-16-5p	*RB1*	2267-2287	0.643	CDS	-15.400	-1.382	0.446	1000	0.754
hsa-miR-130a-3p	*RB1*	3968-3988	0.642	3'UTR	-18.200	-4.887	0.465	0.519	0.558
hsa-miR-130a-3p	*RB1*	1616-1631	0.642	CDS	-16.100	-6.238	0.437	0.939	0.520
hsa-miR-93-5p	*RB1*	3174-3202	0.642	3'UTR	-17.200	-1.315	0.444	0.411	0.121
hsa-let-7a-5p	*RB1*	634-653	0.641	CDS	-16.500	-3.575	0.441	0.994	0.168
hsa-miR-223-3p	*RB1*	1474-1491	0.639	CDS	-17.200	-7.779	0.449	0.840	0.469
hsa-miR-25-3p	*RB1*	2264-2284	0.639	CDS	-16.100	-4.100	0.485	1000	0.753
hsa-let-7a-5p	*RB1*	2060-2071	0.638	CDS	-17.600	-6.107	0.418	0.921	0.680
hsa-let-7a-5p	*RB1*	1854-1876	0.638	CDS	-11.900	4.859	0.404	0.999	0.606
hsa-miR-130a-3p	*RB1*	1514-1526	0.638	CDS	-12.700	-7.242	0.485	0.942	0.484
hsa-miR-142-3p	*RB1*	3456-3475	0.638	3'UTR	-11.400	-3.809	0.520	0.136	0.277
hsa-miR-21-5p	*RB1*	1603-1629	0.638	CDS	-18.200	-6.484	0.411	1000	0.516
hsa-miR-93-5p	*RB1*	1617-1636	0.638	CDS	-15.900	-5.448	0.414	0.948	0.521
hsa-miR-31-5p	*RB1*	1615-1633	0.635	CDS	-17.100	-6.873	0.456	0.946	0.520
hsa-miR-31-5p	*RB1*	1615-1636	0.635	CDS	-17.100	-6.574	0.415	0.953	0.520
hsa-let-7a-5p	*RB1*	2574-2587	0.633	CDS	-13.900	-7.311	0.494	0.997	0.864
hsa-miR-15b-5p	*RB1*	1907-1933	0.633	CDS	-17.700	-0.618	0.493	0.979	0.625
hsa-miR-142-3p	*RB1*	4419-4442	0.632	3'UTR	-12.900	-2.613	0.447	0.393	0.806
hsa-miR-223-3p	*RB1*	1474-1486	0.632	CDS	-15.100	-8.156	0.466	0.779	0.469
hsa-let-7a-5p	*RB1*	4413-4443	0.630	3'UTR	-16.900	-3.973	0.400	0.358	0.803
hsa-miR-31-5p	*RB1*	3937-3971	0.630	3'UTR	-21.800	-4.536	0.435	0.372	0.541
hsa-miR-93-5p	*RB1*	726-761	0.630	CDS	-13.400	0.322	0.492	0.887	0.201
hsa-miR-16-5p	*RB1*	2018-2043	0.629	CDS	-15.400	-2.556	0.506	0.832	0.665
hsa-miR-22-3p	*RB1*	1471-1491	0.628	CDS	-15.600	-4.717	0.422	0.863	0.468
hsa-miR-205-5p	*RB1*	4482-4487	0.626	3'UTR	-15.600	-10.472	0.409	0.010	0.841
hsa-miR-142-3p	*RB1*	4633-4657	0.625	3'UTR	-10.700	1.878	0.457	0.730	0.924

hsa-let-7a-5p	*RB1*	1497-1529	0.624	CDS	-17.700	2.218	0.419	0.977	0.478
hsa-miR-142-3p	*RB1*	3973-3988	0.624	3'UTR	-16.600	-5.175	0.409	0.534	0.561
hsa-miR-223-3p	*RB1*	1617-1630	0.623	CDS	-12.200	-2.811	0.452	0.930	0.521
hsa-miR-31-5p	*RB1*	4544-4569	0.623	3'UTR	-18.300	-1.786	0.438	0.731	0.875
hsa-let-7a-5p	*RB1*	1685-1706	0.622	CDS	-10.400	-5.238	0.521	0.999	0.545
hsa-miR-200c-3p	*RB1*	2202-2223	0.622	CDS	-18.100	-3.304	0.442	1000	0.731
hsa-miR-31-5p	*RB1*	2213-2231	0.620	CDS	-16.900	-0.534	0.508	0.980	0.734
hsa-miR-31-5p	*RB1*	3786-3800	0.618	3'UTR	-21.700	-12.335	0.408	0.136	0.458
hsa-miR-31-5p	*RB1*	2818-2841	0.618	CDS	-15.500	-2.010	0.520	0.998	0.952
hsa-miR-130a-3p	*RB1*	608-640	0.617	CDS	-14.600	2.362	0.428	0.979	0.159
hsa-miR-142-3p	*RB1*	3679-3716	0.617	3'UTR	-16.800	-6.172	0.482	0.143	0.399
hsa-miR-93-5p	*RB1*	4168-4191	0.617	3'UTR	-17.500	-2.711	0.423	0.537	0.668
hsa-miR-142-3p	*RB1*	329-347	0.615	CDS	-12.500	1.314	0.433	1000	0.058
hsa-miR-142-3p	*RB1*	460-484	0.614	CDS	-18.400	0.030	0.453	0.995	0.105
hsa-miR-200c-3p	*RB1*	3178-3198	0.614	3'UTR	-16.300	-0.528	0.475	0.468	0.124
hsa-miR-205-5p	*RB1*	3972-3989	0.614	3'UTR	-16.100	-3.415	0.416	0.520	0.560
hsa-miR-205-5p	*RB1*	2962-2974	0.614	3'UTR	-22.500	-8.945	0.401	0.355	0.005
hsa-miR-25-3p	*RB1*	2962-2983	0.613	3'UTR	-21.900	-9.669	0.482	0.313	0.005
hsa-miR-15b-5p	*RB1*	2962-2980	0.610	3'UTR	-20.500	-8.462	0.502	0.363	0.005
hsa-miR-200c-3p	*RB1*	3956-3997	0.610	3'UTR	-17.800	-4.113	0.508	0.430	0.551
hsa-miR-22-3p	*RB1*	2202-2222	0.610	CDS	-16.700	-1.873	0.426	1.000	0.731
hsa-miR-93-5p	*RB1*	3425-3461	0.610	3'UTR	-15.700	1.115	0.519	0.198	0.259
hsa-miR-15b-5p	*RB1*	3217-3232	0.609	3'UTR	-18.800	-8.093	0.418	0.220	0.145
hsa-miR-200c-3p	*RB1*	3760-3788	0.609	3'UTR	-20.500	-6.738	0.457	0.440	0.444
hsa-miR-200c-3p	*RB1*	3178-3202	0.608	3'UTR	-16.900	-1.870	0.468	0.394	0.124
hsa-let-7a-5p	*RB1*	4035-4058	0.606	3'UTR	-16.600	-4.535	0.611	0.176	0.595
hsa-let-7a-5p	*RB1*	615-649	0.605	CDS	-15.800	-0.430	0.439	0.981	0.161
hsa-miR-200c-3p	*RB1*	3436-3447	0.604	3'UTR	-16.500	-5.723	0.513	0.003	0.266
hsa-miR-31-5p	*RB1*	1339-1373	0.603	CDS	-15.300	4.459	0.407	0.999	0.421
hsa-miR-130a-3p	*RB1*	3211-3230	0.601	3'UTR	-17.400	-5.972	0.472	0.123	0.142
hsa-miR-205-5p	*RB1*	2595-2619	0.599	CDS	-16.200	1.261	0.473	0.999	0.872
hsa-miR-93-5p	*RB1*	913-930	0.597	CDS	-17.200	-7.983	0.518	1000	0.268

hsa-miR-130a-3p	*RB1*	3968-3997	0.596	3'UTR	-15.800	-2.439	0.476	0.424	0.558
hsa-miR-205-5p	*RB1*	2861-2875	0.595	CDS	-15.200	-1.904	0.422	1000	0.967
hsa-miR-93-5p	*RB1*	2964-2981	0.594	3'UTR	-16.800	-7.050	0.523	0.272	0.006
hsa-miR-142-3p	*RB1*	617-641	0.593	CDS	-12.600	3.021	0.455	0.974	0.162
hsa-miR-205-5p	*RB1*	2257-2279	0.593	CDS	-16.300	1.362	0.458	1	0.750
hsa-miR-205-5p	*RB1*	406-423	0.593	CDS	-15.200	-1.979	0.419	1000	0.086
hsa-miR-223-3p	*RB1*	461-484	0.592	CDS	-16.900	-1.212	0.472	0.999	0.106
hsa-let-7a-5p	*RB1*	4035-4053	0.590	3'UTR	-15.700	-3.625	0.521	0.217	0.595
hsa-miR-130a-3p	*RB1*	1934-1970	0.589	CDS	-16.100	1.834	0.632	0.987	0.634
hsa-miR-130a-3p	*RB1*	2848-2869	0.589	CDS	-17.300	-1.530	0.450	0.994	0.962
hsa-miR-200c-3p	*RB1*	2849-2871	0.589	CDS	-15.900	2.852	0.426	0.996	0.963
hsa-miR-93-5p	*RB1*	2964-2984	0.589	3'UTR	-17.900	-8.308	0.498	0.233	0.006
hsa-let-7a-5p	*RB1*	2336-2359	0.588	CDS	-16.400	0.462	0.434	0.955	0.779
hsa-miR-142-3p	*RB1*	2599-2624	0.587	CDS	-17.100	4.340	0.441	0.988	0.873
hsa-let-7a-5p	*RB1*	2054-2068	0.586	CDS	-17.300	-7.499	0.496	0.996	0.677
hsa-miR-93-5p	*RB1*	3683-3717	0.586	3'UTR	-15.800	-5.123	0.466	0.123	0.401
hsa-miR-93-5p	*RB1*	2964-2978	0.585	3'UTR	-16.800	-7.390	0.520	0.326	0.006
hsa-let-7a-5p	*RB1*	2447-2472	0.584	CDS	-12.900	0.673	0.485	1	0.818
hsa-miR-16-5p	*RB1*	4545-4570	0.583	3'UTR	-16.200	2.176	0.422	0.698	0.875
hsa-miR-93-5p	*RB1*	458-478	0.583	CDS	-17.300	5.085	0.429	0.993	0.105
hsa-miR-130a-3p	*RB1*	2965-2980	0.581	3'UTR	-15.700	-6.376	0.541	0.266	0.007
hsa-miR-16-5p	*RB1*	3212-3232	0.581	3'UTR	-16.100	-4.783	0.478	0.170	0.142
hsa-miR-200c-3p	*RB1*	3956-3995	0.581	3'UTR	-15.500	-1.578	0.492	0.449	0.551
hsa-miR-93-5p	*RB1*	3683-3721	0.580	3'UTR	-16.800	-2.799	0.485	0.113	0.401
hsa-miR-200c-3p	*RB1*	3211-3231	0.579	3'UTR	-15.400	-3.979	0.457	0.145	0.142
hsa-miR-93-5p	*RB1*	2448-2473	0.579	CDS	-13.500	3.954	0.452	1	0.819
hsa-let-7a-5p	*RB1*	4470-4503	0.578	3'UTR	-11.700	-1.219	0.465	0.338	0.834
hsa-miR-31-5p	*RB1*	458-481	0.575	CDS	-16.400	6.118	0.461	0.994	0.105
hsa-miR-31-5p	*RB1*	458-477	0.573	CDS	-15.700	6.671	0.401	0.993	0.105
hsa-miR-25-3p	*RB1*	3008-3021	0.568	3'UTR	-15.700	-9.726	0.522	0.001	0.030
hsa-miR-93-5p	*RB1*	3223-3238	0.568	3'UTR	-15.900	-5.506	0.420	0.258	0.148

hsa-miR-130a-3p	*RB1*	3265-3284	0.566	3'UTR	-20.300	-9.043	0.483	0.003	0.172
hsa-miR-22-3p	*RB1*	2015-2051	0.566	CDS	-16.800	1.018	0.467	0.838	0.663
hsa-miR-223-3p	*RB1*	461-493	0.565	CDS	-17.300	-0.046	0.483	0.999	0.106
hsa-let-7a-5p	*RB1*	615-646	0.564	CDS	-13.300	2.064	0.475	0.980	0.161
hsa-miR-142-3p	*RB1*	3930-3953	0.561	3'UTR	-16.700	-2.680	0.424	0.156	0.537
hsa-miR-16-5p	*RB1*	3212-3229	0.560	3'UTR	-15.800	-4.010	0.464	0.110	0.142
hsa-miR-200c-3p	*RB1*	3211-3244	0.558	3'UTR	-17.800	-5.614	0.448	0.127	0.142
hsa-miR-130a-3p	*RB1*	2447-2475	0.555	CDS	-15.200	1.492	0.450	1	0.818
hsa-miR-142-3p	*RB1*	3792-3821	0.555	3'UTR	-18.400	0.193	0.430	0.363	0.461
hsa-miR-130a-3p	*RB1*	2447-2477	0.554	CDS	-16.400	1.886	0.442	1	0.818
hsa-miR-31-5p	*RB1*	3225-3253	0.553	3'UTR	-22.600	-8.754	0.406	0.154	0.150
hsa-miR-31-5p	*RB1*	3225-3240	0.551	3'UTR	-17.900	-6.138	0.408	0.246	0.150
hsa-miR-142-3p	*RB1*	2840-2872	0.549	CDS	-16.400	7.221	0.408	0.995	0.959
hsa-miR-142-3p	*RB1*	1510-1528	0.546	CDS	-11.400	1.477	0.447	0.960	0.482
hsa-let-7a-5p	*RB1*	3015-3042	0.545	3'UTR	-17.500	-3.691	0.408	0.012	0.034
hsa-let-7a-5p	*RB1*	615-643	0.543	CDS	-13.200	2.165	0.473	0.978	0.161
hsa-let-7a-5p	*RB1*	46-62	0.542	5'UTR	-10.500	-5.158	0.650	0.993	0.277
hsa-miR-142-3p	*RB1*	4568-4586	0.542	3'UTR	-12.600	-4.662	0.441	0.343	0.888
hsa-miR-200c-3p	*RB1*	3211-3236	0.539	3'UTR	-16.400	-4.634	0.524	0.161	0.142
hsa-miR-93-5p	*RB1*	2448-2479	0.539	CDS	-15.900	4.024	0.404	1	0.819
hsa-let-7a-5p	*RB1*	3271-3296	0.538	3'UTR	-18.200	-3.875	0.451	0.371	0.175
hsa-miR-142-3p	*RB1*	3211-3229	0.536	3'UTR	-13.200	-1.321	0.456	0.107	0.142
hsa-miR-205-5p	*RB1*	2811-2845	0.536	CDS	-16.400	6.842	0.417	0.977	0.949
hsa-miR-205-5p	*RB1*	2654-2687	0.533	CDS	-15.400	6.137	0.424	0.995	0.893
hsa-miR-21-5p	*RB1*	3022-3047	0.532	3'UTR	-15.100	-1.893	0.453	0.013	0.038
hsa-miR-93-5p	*RB1*	3209-3234	0.528	3'UTR	-15.500	-3.013	0.492	0.165	0.141
hsa-let-7a-5p	*RB1*	2976-2989	0.525	3'UTR	-17.100	-7.808	0.434	0.109	0.013
hsa-let-7a-5p	*RB1*	3268-3283	0.523	3'UTR	-17.200	-8.679	0.500	0.002	0.173
hsa-miR-16-5p	*RB1*	2267-2302	0.523	CDS	-16.100	12.116	0.407	1.000	0.754
hsa-miR-22-3p	*RB1*	906-930	0.523	CDS	-16.600	-0.726	0.489	1000	0.266
hsa-miR-142-3p	*RB1*	4568-4584	0.520	3'UTR	-11.600	-4.031	0.490	0.266	0.888
hsa-let-7a-5p	*RB1*	3114-3146	0.519	3'UTR	-10.100	2.533	0.484	0.106	0.089

hsa-let-7a-5p	*RB1*	2223-2249	0.516	CDS	-15.300	-2.673	0.426	0.986	0.738
hsa-miR-16-5p	*RB1*	93-106	0.511	5'UTR	-21.300	-16.200	0.564	0.999	0.560
hsa-miR-200c-3p	*RB1*	3266-3281	0.509	3'UTR	-18.900	-10.201	0.492	0.002	0.172
hsa-miR-200c-3p	*RB1*	163-175	0.508	5'UTR	-18.400	-9.111	0.518	0.986	0.982
hsa-let-7a-5p	*RB1*	3217-3235	0.506	3'UTR	-12.400	-1.748	0.482	0.216	0.145
hsa-miR-15b-5p	*RB1*	2999-3020	0.504	3'UTR	-19.400	-8.500	0.454	0.001	0.025
hsa-miR-130a-3p	*RB1*	3211-3232	0.501	3'UTR	-11.300	0.290	0.470	0.164	0.142

* Start and end position of the target region (site) predicted to be bound by miRNA; [a] Probability of the site being an miRNA binding site as predicted by our nonlinear logistic model; [b] A measure of stability for miRNA:target hybrid as computed by RNAhybrid; [c] A measure of the total energy change of the hybridization; [d] A measure of structural accessibility as computed by the average probability of a nucleotide being single-stranded (i.e., unpaired) for the nucleotides in the predicted binding site; [e] Conservation score by the PhastCons program for the binding site; [j] Relative starting location of the predicted binding site along the length of the sequence (e.g., for 3″ UTR, 0 indicates the 5″ end of the UTR, and 1 corresponds to the 3″ end).

Supplementary Table S3. All pathways involving *TP53* and *RB1* genes and the upregulated miRNAs that regulate them.

KEGG Pathway	p-Value pathway	miRNAs Name	Gene Name
Viral carcinogenesis (hsa05203)	$1.16x10^{-10}$	let-7a-5p<br>miR-16-5p<br>miR-205-5p<br>miR-22-3p<br>miR-93-5p<br>miR-130a-3p	*TP53* and *RB1*<br>*TP53*<br>*TP53*<br>*TP53*<br>*TP53* and *RB1*<br>*RB1*
Chronic myeloid leukemia (hsa05220)	0.0005998	let-7a-5p<br>miR-16-5p<br>miR-205-5p<br>miR-22-3p<br>miR-93-5p<br>miR-130a-3p	*TP53* and *RB1*<br>*TP53*<br>*TP53*<br>*TP53*<br>*TP53* and *RB1*<br>*RB1*
Central carbon metabolism in cancer (hsa05230)	0.0011443	let-7a-5p<br>miR-16-5p<br>miR-205-5p<br>miR-22-3p<br>miR-93-5p	*TP53* and *RB1*<br>*TP53*<br>*TP53*<br>*TP53*<br>*TP53* and *RB1*
Glioma (hsa05214)	0.0017756	let-7a-5p<br>miR-16-5p<br>miR-205-5p<br>miR-22-3p<br>miR-93-5p<br>miR-130a-3	*TP53* and *RB1*<br>*TP53*<br>*TP53*<br>*TP53*<br>*TP53* and *RB1*<br>*RB1*
Cell cycle (hsa04110)	0.0038093	let-7a-5p<br>miR-16-5p<br>miR-205-5p<br>miR-22-3p<br>miR-93-5p<br>miR-130a-3	*TP53* and *RB1*<br>*TP53*<br>*TP53*<br>*TP53*<br>*TP53* and *RB1*<br>*RB1*
Melanoma (hsa05218)	0.0041201	let-7a-5p<br>miR-16-5p	*TP53* and *RB1*<br>*TP53*

		miR-205-5p	*TP53*
		miR-22-3p	*TP53*
		miR-93-5p	*TP53* and *RB1*
		miR-130a-3	*RB1*
Bladder cancer (hsa05219)	0.0183585	let-7a-5p	*TP53* and *RB1*
		miR-16-5p	*TP53*
		miR-205-5p	*TP53*
		miR-22-3p	*TP53*
		miR-93-5p	*TP53* and *RB1*
		miR-130a-3	*RB1*
Endometrial cancer (hsa05213)	0.0309891	let-7a-5p	*TP53* and *RB1*
		miR-16-5p	*TP53*
		miR-205-5p	*TP53*
		miR-22-3p	*TP53*
		miR-93-5p	*TP53* and *RB1*
		miR-130a-3	*RB1*
Hepatitis B (hsa05161)	0.0313645	let-7a-5p	*TP53* and *RB1*
		miR-16-5p	*TP53*
		miR-205-5p	*TP53*
		miR-22-3p	*TP53*
		miR-93-5p	*TP53* and *RB1*
		miR-130a-3	*RB1*
Non-small cell lung cancer (hsa05223)	0.0380989	let-7a-5p	*TP53* and *RB1*
		miR-16-5p	*TP53*
		miR-205-5p	*TP53*
		miR-22-3p	*TP53*
		miR-93-5p	*TP53* and *RB1*
		miR-130a-3	*RB1*
Pancreatic cancer (hsa05212)	0.0478991	let-7a-5p	*TP53* and *RB1*
		miR-16-5p	*TP53*
		miR-205-5p	*TP53*
		miR-22-3p	*TP53*
		miR-93-5p	*TP53* and *RB1*
		miR-130a-3	*RB1*

Small cell lung cancer (hsa05222)	0.0479915	let-7a-5p	*TP53* and *RB1*
		miR-16-5p	*TP53*
		miR-205-5p	*TP53*
		miR-22-3p	*TP53*
		miR-93-5p	*TP53* and *RB1*
		miR-130a-3	*RB1*
Prostate cancer (hsa05215)	0.0492142	let-7a-5p	*TP53* and *RB1*
		miR-16-5p	*TP53*
		miR-205-5p	*TP53*
		miR-22-3p	*TP53*
		miR-93-5p	*TP53* and *RB1*
		miR-130a-3	*RB1*
